# Antidepressant use and cognitive decline in patients with dementia: a national cohort study

**DOI:** 10.1186/s12916-025-03851-3

**Published:** 2025-02-25

**Authors:** Minjia Mo, Tamar Abzhandadze, Minh Tuan Hoang, Simona Sacuiu, Pol Grau Jurado, Joana B. Pereira, Luana Naia, Julianna Kele, Silvia Maioli, Hong Xu, Maria Eriksdotter, Sara Garcia-Ptacek

**Affiliations:** 1 Division of Clinical Geriatrics, Department of Neurobiology, Care Sciences and Society, Karolinska Institutet, Blickagången 16, Stockholm, 14152 Sweden; 2 Department of Occupational Therapy and Physiotherapy, Sahlgrenska University Hospital, Rehabiliteringsmedicin, Vita Stråket 12, Vån 4, Gothenburg, 41345 Sweden; 3 Department of Medical Epidemiology and Biostatistics, Karolinska Institutet, Nobels Väg 12a, Stockholm, 17165 Sweden; 4 Department of Neuropsychiatry, Sahlgrenska University Hospital Mölndal, Region Västra Götaland, Sweden, Wallinsgatan 6, Mölndal, 43141 Sweden; 5 Department of Psychiatry and Neurochemistry, Institute of Neuroscience and Physiology, Neuropsychiatric Epidemiology (EPINEP), Sahlgrenska Academy, University of Gothenburg, Medicinaregatan 3, Göteborg, 413 90 Sweden; 6 Theme Inflammation and Aging, Medical Unit Aging, Karolinska University Hospital, Karolinska Vägen 37A, Stockholm, 171 64 Sweden; 7 Department of Clinical Neurosciences, Karolinska Institutet, Nobels Väg 9, Stockholm, D3, 17165 Sweden; 8 Department of Laboratory Medicine, Team Neurovascular Biology and Health, Clinical Immunology, Karolinska Institutet, H5 Laboratoriemedicin, H5 Klin Immunologi Bergman, Huddinge, 14152 Sweden

**Keywords:** Dementia, Antidepressants, Cognitive decline, Mortality, Cohort

## Abstract

**Background:**

Dementia is associated with psychiatric symptoms but the effects of antidepressants on cognitive function in dementia are understudied. We aimed to investigate the association between antidepressants and cognitive decline in patients with dementia, and the risk of severe dementia, fractures and death, depending on antidepressant class, drug, and dose.

**Methods:**

This is a national cohort study. Patients with dementia registered in the Swedish Registry for Cognitive/Dementia Disorders-SveDem from May 1, 2007, until October 16, 2018, with at least one follow-up after dementia diagnosis, and who were new users of antidepressants, were included. Antidepressant use as a time varying exposure defined during the 6 months leading up to dementia diagnosis or each subsequent follow-up. We used linear mixed models to examine the association between antidepressant use and cognitive trajectories assessed by Mini-Mental State Examination (MMSE) scores. We used Cox proportional hazards models to calculate the hazard ratios for severe dementia (MMSE score < 10), fracture, and death. We compared antidepressant classes and drugs, and analyzed dose–response.

**Results:**

We included 18740 patients (10 205 women [54.5%]; mean [SD] age, 78.2[7.4] years), of which 4271 (22.8%) received at least one prescription for an antidepressant. During follow-up, a total of 11912 prescriptions for antidepressants were issued, with selective serotonin reuptake inhibitors (SSRI) being the most common (64.8%). Antidepressant use was associated with faster cognitive decline (*β* (95% CI) = − 0.30(− 0.39, − 0.21) points/year), in particular sertraline (− 0.25(− 0.43, − 0.06) points/year), citalopram (− 0.41(− 0.55, − 0.27) points/year), escitalopram (− 0.76(− 1.09, − 0.44) points/year), and mirtazapine (− 0.19(− 0.34, − 0.04) points/year) compared with non-use. The association was stronger in patients with severe dementia (initial MMSE scores 0–9). Escitalopram showed a greater decline rate than sertraline. Compared with non-use, dose response of SSRIs on greater cognitive decline and higher risks of severe dementia, all-cause mortality, and fracture were observed.

**Conclusions:**

In this cohort study, current antidepressant use was associated with faster cognitive decline; furthermore, higher dispensed doses of SSRIs were associated with higher risk for severe dementia, fractures, and all-cause mortality. These findings highlight the significance of careful and regular monitoring to assess the risks and benefits of different antidepressants use in patients with dementia.

**Supplementary Information:**

The online version contains supplementary material available at 10.1186/s12916-025-03851-3.

## Background

Antidepressants are widely used in patients with dementia to improve neuropsychiatric symptoms, such as anxiety, depression, aggression, and sleep disorders [1]. Selective serotonin reuptake inhibitors (SSRIs) and serotonin norepinephrine reuptake inhibitors (SNRIs) are considered first-line pharmacotherapy for depression due to fewer side effects compared to other classes of antidepressants. However, older adults with depression receiving SSRIs/SNRIs were associated with an increased risk of dementia compared to psychotherapy [2].

The clinical efficacy of antidepressants on dementia progression is uncertain. Tricyclic antidepressants (TCAs) are anticholinergics and negatively impact cognition [3]. In contrast, a beneficial impact of SSRIs on neurogenesis and pathologic biomarkers, including amyloid burden and tau deposits, has been reported [4, 5], along with evidence suggesting they may delay the progression from mild cognitive impairment (MCI) to Alzheimer’s dementia (AD) [6, 7] among persons with depression. The different cognitive effects of antidepressant classes may be due to the different mechanisms of action, driven by how these drugs proximally act on various neurotransmitters in the brain [3]. Based on the national guidelines for care of depression and anxiety syndrome in Sweden, sertraline and escitalopram form the first-line of therapy for older patients [8]. However, antidepressants do not seem to work as well in people with dementia, and dysfunctions of cognitive control in dementia appears to decrease the effectiveness of some SSRIs [9].

Findings from observational studies investigating the impact of antidepressant use on cognitive outcomes are inconclusive. Some longitudinal data observed slower declines in cognition with antidepressant use in patients with AD [10, 11], while most of the cohort studies found no evidence for an association between antidepressant use and post-treatment cognitive decline [12–15], and negative associations of antidepressant use with subsequent cognitive impairment has been reported [16, 17]. Previous analyses were limited to the period before dementia diagnosis [15], a single population, such as patients with depression but not in patients with dementia [12, 14, 16], a short-term follow-up ^11^ [13], and reporting the effect of antidepressants on cognition as a secondary outcome [17]. There is a gap in understanding how different antidepressant classes, specific drugs, and doses affect the progression of cognitive decline in patients with dementia. Moreover, existing studies have not thoroughly explored how individual factors, such as age, sex, and dementia severity, may modify the association between antidepressant use and cognitive outcomes.

Some antidepressants have anticholinergic activities which could contribute to worse cognition, and potentially, falls and mortality, particularly in dementia types with cholinergic deficit such as AD and dementia with Lewy bodies (DLB) [18–20]. Moreover, previous studies were conducted mainly in patients with AD; findings related to other dementias, such as vascular dementia (VaD) [21], frontotemporal dementia (FTD) [22], DLB [23], and Parkinson’s disease dementia (PDD) are scarce. Long-term follow-up studies are difficult due to high attrition rates and loss of follow-up [24]. Currently, direct comparisons of different individual drugs on cognitive decline in patients with dementia are lacking.

This study examines the long-term effect of antidepressants on cognitive decline, fractures, and mortality in patients with dementia. It aims to address the knowledge gap by providing a comprehensive analysis of how different antidepressant classes, drugs, and doses impact dementia progression, and whether factors like dementia subtypes, severity, and medications modify these effects. The findings may inform clinical care and contribute to future research, providing insights for primary care practitioners and specialists involved in dementia healthcare.

## Methods

### Study design and data source

Based on nationwide Swedish registers, we conducted a population-based cohort study between May 1, 2007, and October 16, 2018. Using the Swedish Registry for Cognitive/Dementia Disorders (SveDem), we identified patients with incident diagnosed dementia. SveDem is a quality registry established in 2007 aiming to register and follow-up all patients with incident dementia in Sweden. SveDem includes patients with incident dementia diagnosis from either primary care or specialist memory clinics and contains information on demographics, diagnostic process, and cognitive and mortality outcomes. For this study, we used the type of dementia, cognitive evaluation by MMSE, coresident status, and type of diagnostic unit from SveDem [25]. The National Patient Register contains nationwide records on inpatient care since 1987 and more than 80% of specialized (hospital-based) outpatient care since 2001 [26]. The Swedish Prescribed Drug Register provides complete data on dispensation of prescription medications from all pharmacies since July 2005 [27]. The Cause of Death Registry contains data on overall and specific mortality and dates of death [28]. The Swedish unique personal identity number was used to identify patients across sources and to merge data.

### Study population

The study population included patients with incident dementia registered in SveDem between May 1, 2007, and October 16, 2018. We defined index date as the date of the dementia diagnosis in SveDem. We excluded patients if any of the following applied: missing information on MMSE score at baseline; a record of antidepressant dispensation prior to the 6-month period before the date of dementia diagnosis or no follow-ups. To permit comparisons between drugs, we also excluded patients who received prescriptions for different antidepressants within a class or antidepressants from different classes in the same 6-month period. Additional file 1: Figure S1 presents a flowchart for the selection of participants.

Dementia disorders are clinically diagnosed and recorded according to the International Classification of Diseases, Tenth Revision (ICD-10) codes [29], with the McKeith criteria [30] used for DLB, the Lund-Manchester criteria [31] for FTD, and the Movement Disorder Society Task Force criteria [32] for PDD, respectively (Additional file 2: Table S1). Dementia was defined at the time of the establishment of the dementia diagnosis, coded as AD, mixed dementia, VaD, DLB, FTD, PDD, and other dementias, which included unspecified dementia and other dementia types not classified above. DLB and PDD share pathological and clinical characteristics, are considered part of a continuum within the spectrum of LBD [33, 34], and were therefore merged for this study as LBDs.

### Antidepressant exposure

We treated antidepressant use as a time varying exposure and defined it as a dispensation of medication during the 6 months leading up to the dementia diagnosis or each subsequent follow-up date through the Prescribed Drug Register by ATC codes (Additional file 2: Table S2). This is followed by grouping of antidepressant drugs into five classes according to the mode of action: SSRIs, SNRIs, TCAs, and other antidepressants.

We extracted information on the dose of each dispensation of antidepressants within the 6-month period preceding each SveDem entry date or each subsequent follow-up date. Cumulative doses dispensed during these time periods were expressed as number of defined daily doses (DDD) in each package or dispensation. The DDD for each medication is defined by the World Health Organization [35] and was used to allow dose comparisons between different medications. When multiple dispensations were used, their DDD were added [36].

### Outcomes

The main outcome for this study was cognitive decline, defined as MMSE score change over the years. Information of baseline and follow-up MMSE scores were collected from SveDem. The secondary outcomes included severe dementia, fracture, and death. Severe dementia was specifically defined as MMSE score < 10 during follow-up [37]. Information of death from all causes was extracted from the Cause of Death Registry based on ICD-10 codes. Data on fractures, defined as any fractures occurring at skeletal sites during follow-up, were extracted from the National Patient Register and Cause of Death Registry by using the ICD-10 codes (Additional file 2: Table S3). Patients were observed from index date through October 16, 2018, for any secondary outcomes.

### Covariates

We defined covariates at the index date, including age, sex, coresident status, type of diagnostic unit, and calendar year of diagnosis. We used ICD-10 codes to identify common and major physical disorders diagnosed within 3 years before the date of dementia diagnosis from the National Patient Register (Additional file 2: Table S3). We used the Charlson Comorbidity Index score [38] (CCIs) to assess medical comorbidities, using a weighted sum of diagnosed chronic disorders [39] but excluding dementia. In addition, we also included depression and fracture diagnosed within 3 years before the date of dementia diagnosis (Additional file 2: Table S3).

Data on medications were ascertained by ATC codes and defined as the presence of filled pharmacy prescriptions within the 6 months prior to and at the date of dementia diagnosis, including angiotensin-converting enzyme inhibitors/angiotensin receptor blockers, β-blocking agents, calcium channel blockers, nonsteroidal anti-inflammatory drugs, diuretics, lipid-modifying agents, antiplatelets, antipsychotics, anxiolytics, and hypnotics (Additional file 2: Table S4). Anti-dementia medications (i.e., cholinesterase inhibitors [ChEIs] and memantine) use was defined as a dispensation of medication during the 6 months after dementia diagnosis (Additional file 2: Table S4).

### Statistical analysis

We used linear mixed models to examine the associations between the use of antidepressants and cognitive decline (MMSE score change) over the years. We treated antidepressant use as a time varying exposure to account for patients stopping treatment and changing between treatments during follow-up. We considered patients to be exposed to treatment if no gaps of more than 90 days existed between the end of one prescription and the start of the next. Follow-up time was treated as a continuous measure defined as years since first cognitive assessment. The model included antidepressant use and follow-up time and an interaction between drug use and time. Following our previous work in SveDem [36], a linear trend over time was assumed and the model allowed for a random intercept and random slope for each patient. We also included the inverse probability of censoring weighting considering the potential effects of dropout or to the presence of a competing risk before the end of follow-up such as death [40].

In addition to examining any dementias, we separately examined the effects for different subtypes of dementia (AD and mixed, VaD, LBD, FTD, and other dementias). The beta coefficients (β) and 95% confidence intervals (CIs) were obtained from adjusted models including age, sex, calendar year of dementia diagnosis, the type of dementia, MMSE score at diagnosis, coresident status, type of diagnostic unit, depression, fracture, CCIs, and medications. For patients diagnosed with AD, mixed dementia, and LBD, we further adjusted for ChEIs and memantine.

The analysis was initially conducted for any antidepressant, followed by each class of antidepressants (SSRIs, SNRIs, TCAs, and other antidepressants) compared with no current treatment. We compared use of individual antidepressant drugs with no current treatment when numbers were sufficient. In addition, different antidepressant classes and individual drugs were directly compared. We investigated the impact of antidepressant dosage by examining it as a continuous variable and categorizing it into three groups within each class (≤ 0.5 DDD, > 0.5/ ≤ 1.0 DDD, > 1.0DDD). Given that 94.8% of patients prescribed TCAs were using a low dose (≤ 0.5 DDD) (Additional file 2: Table S5), the analysis comparing SSRIs and TCAs, specifically citalopram (SSRI) and amitriptyline (TCA), was performed within the low-dose group (≤ 0.5 DDD).

We did subgroup analysis to explore the potential effects of age (median < 78, ≥ 78 years), sex (male/female), coresident status (cohabiting or living alone), type of diagnostic unit (specialist care or primary care), living in nursing home, calendar year of diagnosis (2007–2012, 2013–2018), baseline MMSE score (0–9, 10–19, 20–24, 25–30) and current use of antipsychotics, anxiolytics and hypnotics, on the association between antidepressant use and cognitive decline. Interaction tests between these factors and antidepressant use were conducted separately to assess possible modifying effects. For patients diagnosed with AD and mixed dementia, and LBD, we also conducted stratified analysis by current use of ChEIs and memantine.

We estimated crude incidence rates per 1000 person-years for fracture and all-cause death in all patients at the end of follow-up, and severe dementia in patients who had baseline MMSE score > 10. We used time-dependent Cox proportional hazards models to estimate the associations between time-varying antidepressants and each outcome and calculated the hazard ratios (HRs) with 95% confidence intervals (CIs). We used time since cohort entry as the underlying timescale. Considering the high mortality of patients with dementia after diagnosis, we applied a competing risk model for outcomes of severe dementia and fracture.

Since depression itself has been linked with dementia and cognitive impairment [41, 42], we did a sensitivity analysis among patients without depression to reduce the indication bias. Furthermore, to avoid floor effects, we did a sensitivity analysis among patients with MMSE ≥ 10 and without depression, excluding patients with baseline severe dementia.

Statistical analyses were conducted with SAS version 9.4 (SAS Institute Inc, Cary, NC) and R 4.2.1, with statistical tests using a 2-tailed *P* < 0.05 as the level of statistical significance.

## Results

### Patient characteristics

In total, 18,740 patients were included in this cohort study, 10,205 (54.5%) were women. The mean (SD) age and MMSE score at baseline were 78.2 (7.4) years and 22.1 (4.3), respectively (Additional file 2: Table S6). The total number of person-years of follow-up was 80,737, with a mean (SD) of 4.3 (2.2) years per patient.

### Patterns of antidepressant treatment

During follow-up, a total of 11,912 prescriptions for antidepressants were issued, 4271 (22.8%) patients received at least one prescription for an antidepressant. SSRIs were the most commonly prescribed class, accounting for 64.8%, followed by TCAs (2.2%), SNRIs (2.0%), and the group of other antidepressants (31.0%) (Additional file 1: Figure S2 and Additional file 2: Table S5). The six most commonly prescribed antidepressant drugs comprised 99.0% (*n* = 11 788) of all prescriptions, including citalopram (SSRI), mirtazapine (other), sertraline (SSRI), escitalopram (SSRI), amitriptyline (TCA), and venlafaxine (SNRI), were analyzed separately in some analysis.

### Antidepressant and cognitive decline by dementia subtypes

**Fig. 1:**
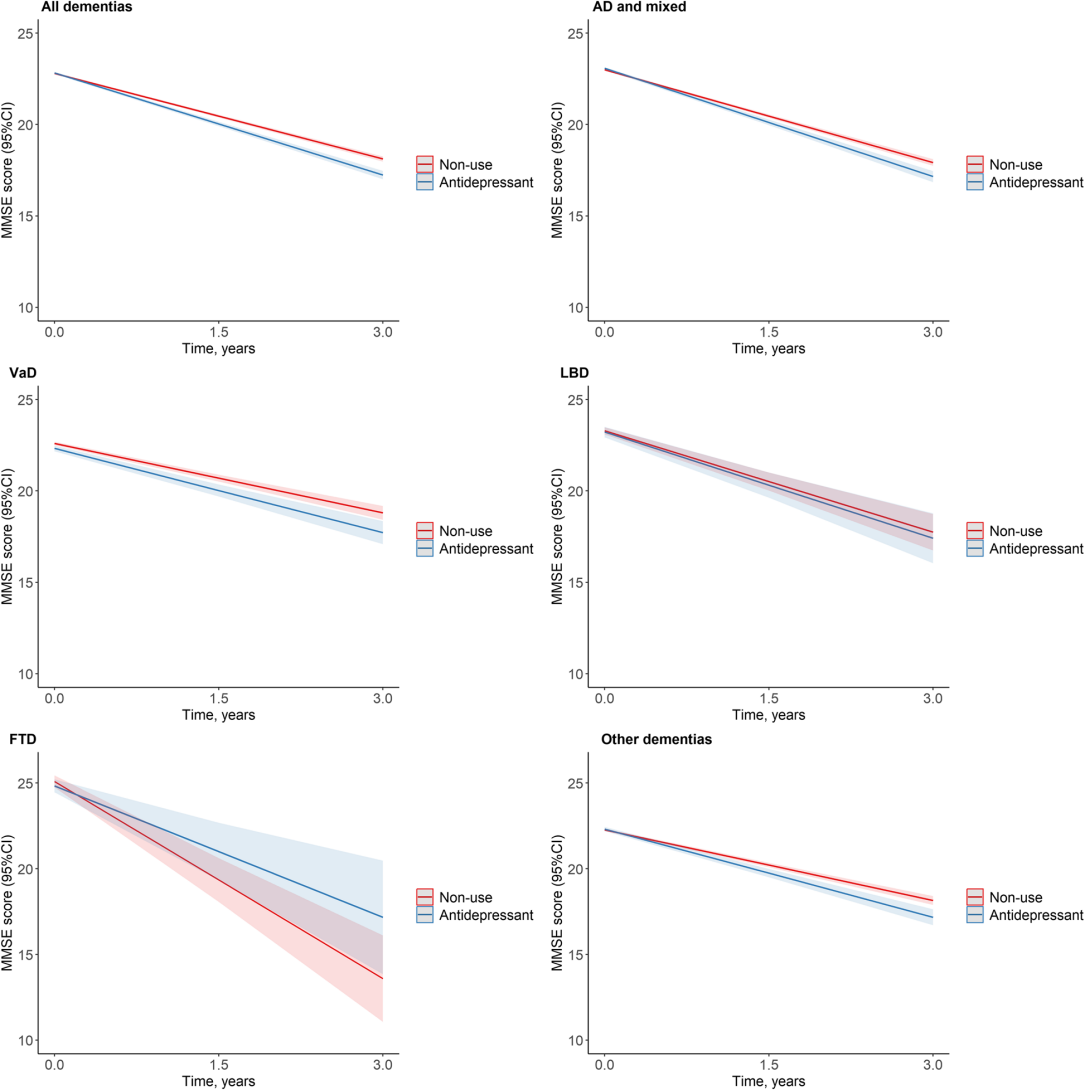
Estimated MMSE trajectories between use of antidepressants and non-use by dementia subtypes^a^. Abbreviations: SveDem, the Swedish Registry for Cognitive/Dementia Disorders; CIs, confidence intervals; MMSE, Mini-Mental State Examination; AD, Alzheimer’s disease; Mixed, mixed dementia; VaD, vascular dementia; LBD, Parkinson’s disease with dementia and dementia with Lewy bodies; FTD, frontotemporal dementia.^a^, Estimated MMSE trajectories from mixed model, adjusted for age, sex, calendar year of diagnosis, the type of dementia, MMSE score at diagnosis, coresident status, care unit, depression, fracture, Charlson Comorbidity Index score , medications (angiotensin-converting enzyme inhibitors (ACEIs)/angiotensin receptor blockers (ARBs), β-blocking agents, calcium channel blockers, nonsteroidal anti-inflammatory drugs, diuretics, lipid-modifying agents, antiplatelets, antipsychotics, anxiolytics, and hypnotics). For patients with AD and mixed dementia and LBD, models further adjusted for cholinesterase inhibitors and memantine

Compared with non-use, antidepressant use was associated with faster cognitive decline (*β* = − 0.30 points/year; 95% CI, − 0.39 to − 0.21) during follow-up (Fig. 1 and Table 1). The results were largely similar for AD and mixed dementia (*β* = − 0.28 points/year; 95% CI, − 0.40 to − 0.16), VaD (*β* = − 0.27 points/year; 95% CI, − 0.52 to − 0.02), and other dementias (*β* = − 0.34 points/year; 95% CI, − 0.52 to − 0.16), except LBD and FTD (Fig. 1 and Table 1).

**Table 1:** Antidepressants and cognitive decline by dementia subtypes

Dementia disorders	*β* (95%CIs)	*P*
All dementias (*n* = 18,740)	− 0.30 (− 0.39, − 0.21)	< .001
AD and mixed (*n* = 11,415)^a^	− 0.28 (− 0.40, − 0.16)	< .001
VaD (*n* = 2194)	− 0.27 (− 0.52, − 0.02)	0.037
LBD (*n* = 587)^a^	− 0.09 (− 0.65, 0.47)	0.759
FTD (*n* = 201)	1.28 (− 0.11, 2.66)	0.072
Other dementias (*n* = 4343)	− 0.34 (− 0.52, − 0.16)	< .001

Adjusted for age, sex, calendar year of diagnosis, the type of dementia, Mini-Mental State Examination score at diagnosis, coresident status, care unit, depression, fracture, Charlson Comorbidity Index score, medications (angiotensin-converting enzyme inhibitors (ACEIs)/angiotensin receptor blockers (ARBs), β-blocking agents, calcium channel blockers, nonsteroidal anti-inflammatory drugs, diuretics, lipid-modifying agents, antiplatelets, antipsychotics, anxiolytics, and hypnotics)

*Abbreviations:* *SveDem* the Swedish Registry for Cognitive/Dementia Disorders, *CIs* Confidence intervals, *AD* Alzheimer’s disease, *Mixed* Mixed dementia, *VaD* Vascular dementia, *LBD* Parkinson’s disease with dementia and dementia with Lewy bodies, *FTD* Frontotemporal dementia

^a^Further adjusted for cholinesterase inhibitors and memantine

### Antidepressant and cognitive decline by antidepressant class and individual drugs

**Fig. 2:**
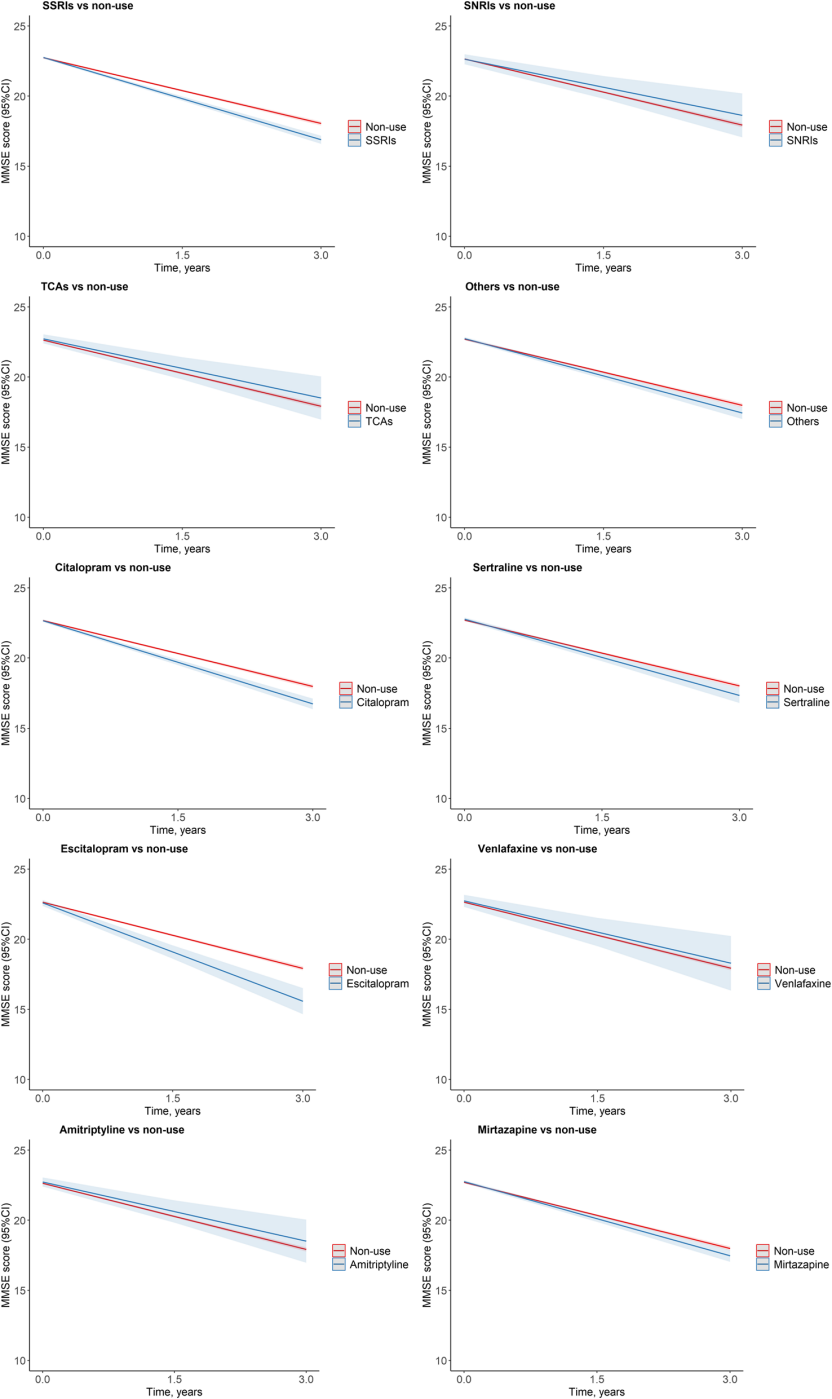
Estimated MMSE trajectories between use of antidepressants and non-use by antidepressant class and individual drugs^a^. Abbreviations: SveDem, the Swedish Registry for Cognitive/Dementia Disorders; CIs, confidence intervals; MMSE, Mini-Mental State Examination; SSRIs, selective serotonin reuptake inhibitors; SNRIs, serotonin and norepinephrine reuptake inhibitors; TCAs, Tricyclic antidepressants; Others, other antidepressants. ^a^, Estimated MMSE trajectories from mixed model, adjusted for age, sex, calendar year of diagnosis, the type of dementia, MMSE score at diagnosis, coresident status, care unit, depression, fracture, Charlson Comorbidity Index score, medications (angiotensin-converting enzyme inhibitors (ACEIs)/angiotensin receptor blockers (ARBs), β-blocking agents, calcium channel blockers, nonsteroidal anti-inflammatory drugs, diuretics, lipid-modifying agents, antiplatelets, antipsychotics, anxiolytics, and hypnotics)

We also observed similar significant results for antidepressant classes for SSRIs (*β* = − 0.39 points/year; 95% CI, − 0.50 to − 0.28) and other antidepressants (*β* = − 0.20 points/year; 95% CI, − 0.35 to − 0.05) and individual antidepressant drugs for sertraline (SSRI) (*β* = − 0.25 points/year; 95% CI, − 0.43 to − 0.06), citalopram (SSRI) (*β* = − 0.41 points/year; 95% CI, − 0.55 to − 0.27), escitalopram (SSRI) (*β* = − 0.76 points/year; 95% CI, − 1.09 to − 0.44), and mirtazapine (other) (*β* = − 0.19 points/year; 95% CI, − 0.34 to − 0.04) (Fig. 2 and Table 2).

**Table 2:** Associations of antidepressants with cognitive decline by antidepressant class and individual drugs

Antidepressant class	*β* (95%CIs)	*P*
Non-use	Ref	
SSRIs	− 0.39 (− 0.50, − 0.28)	< .001
SNRIs	0.24 (− 0.29, 0.77)	0.381
TCAs	0.16 (− 0.36, 0.68)	0.543
Others	− 0.20 (− 0.35, − 0.05)	0.011
Non-use	Ref	
Citalopram (SSRI)	− 0.41 (− 0.55, − 0.27)	< .001
Sertraline (SSRI)	− 0.25 (− 0.43, − 0.06)	0.011
Escitalopram (SSRI)	− 0.76 (− 1.09, − 0.44)	< .001
Venlafaxine (SNRI)	0.09 (− 0.56, 0.74)	0.791
Amitriptyline (TCA)	0.16 (− 0.36, 0.68)	0.543
Mirtazapine (Other)	− 0.19 (− 0.34, − 0.04)	0.014
SSRIs	Ref	
SNRIs	0.43 (− 0.10, 0.96)	0.114
TCAs^a^	0.29 (− 0.27, 0.85)	0.316
Others	0.18 (0.01, 0.34)	0.035
Sertraline (SSRI)	Ref	
Citalopram (SSRI)	0.28 (0.17, 0.39)	< .001
Escitalopram (SSRI)	− 0.51 (− 0.89, − 0.12)	0.01
Venlafaxine (SNRI)	0.14 (− 0.49, 0.77)	0.665
Amitriptyline (TCA)^a^	− 0.01 (− 0.69, 0.68)	0.986
Mirtazapine (Other)	0.08 (− 0.16, 0.31)	0.509

Adjused for age, sex, calendar year of diagnosis, the type of dementia, Mini-Mental State Examination score at diagnosis, coresident status, care unit, depression, fracture, Charlson Comorbidity Index score, medications (angiotensin-converting enzyme inhibitors (ACEIs)/angiotensin receptor blockers (ARBs), β-blocking agents, calcium channel blockers, nonsteroidal anti-inflammatory drugs, diuretics, lipid-modifying agents, antiplatelets, antipsychotics, anxiolytics, and hypnotics)

*Abbreviations:* *SveDem* the Swedish Registry for Cognitive/Dementia Disorders, *CIs* Confidence intervals, *SSRIs* Selective serotonin reuptake inhibitors, *SNRIs* Serotonin and norepinephrine reuptake inhibitors, *TCAs* Tricyclic antidepressants; *Others* other antidepressants

^a^Compared between SSRIs and TCAs, between Sertraline (SSRI) and Amitriptyline (TCA) in low dose group (≤ 0.5 DDD)

**Fig. 3:**
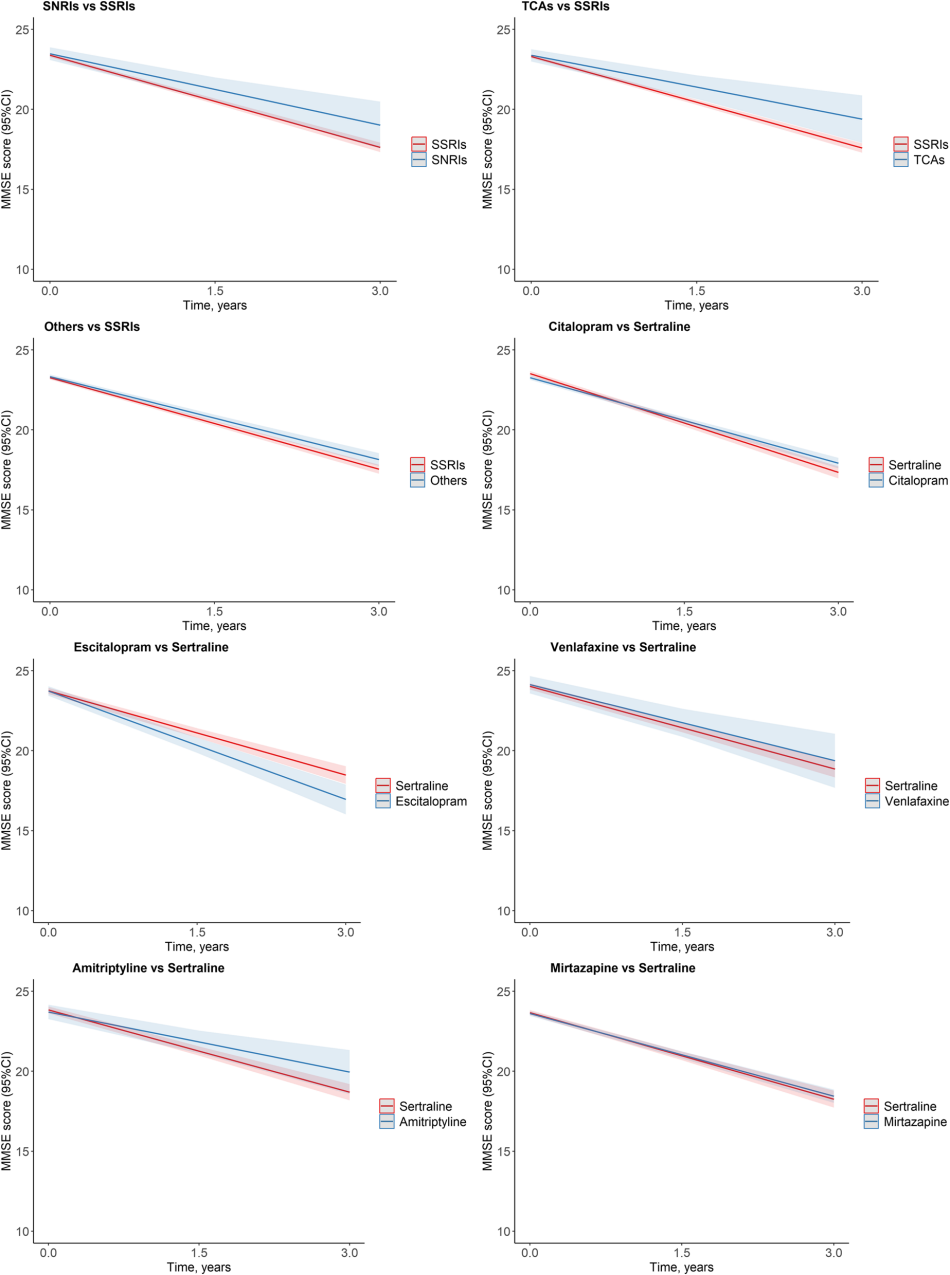
Estimated MMSE trajectories across antidepressant classes and individual drugs^a^. Abbreviations: SveDem, the Swedish Registry for Cognitive/Dementia Disorders; CIs, confidence intervals; MMSE, Mini-Mental State Examination; SSRIs, selective serotonin reuptake inhibitors; SNRIs, serotonin and norepinephrine reuptake inhibitors; TCAs, Tricyclic antidepressants; Others, other antidepressants. ^a^, Estimated MMSE trajectories from mixed model, adjusted for age, sex, calendar year of diagnosis, the type of dementia, MMSE score at diagnosis, coresident status, care unit, depression, fracture, Charlson Comorbidity Index score, medications (angiotensin-converting enzyme inhibitors (ACEIs)/angiotensin receptor blockers (ARBs), β-blocking agents, calcium channel blockers, nonsteroidal anti-inflammatory drugs, diuretics, lipid-modifying agents, antiplatelets, antipsychotics, anxiolytics, and hypnotics)

In addition, compared with sertraline (SSRI), escitalopram (SSRI) (*β* = − 0.51 points/year; 95% CI, − 0.89 to − 0.12) presented faster cognitive decline, while citalopram (SSRI) (*β* = 0.28 points/year; 95% CI, 0.17 to 0.39) showed slower cognitive decline (Fig. 3 and Table 2).

### Antidepressant and cognitive decline by antidepressant dose

Higher dispensed doses of antidepressants, specifically for SSRIs and the group of other antidepressants were associated with faster cognitive decline during follow-up (Table 3 and Additional file 1: Figure S3). Similar results were observed in sensitivity analysis among patients diagnosed with AD and mixed dementia, patients without depression or patients with baseline MMSE ≥ 10 and without depression (Additional file 1: Figure S3 and Additional file 2: Table S7-S10).

**Table 3:** Associations of antidepressant dose with cognitive decline

Antidepressant class and dose category	*β* (95%CIs)			
	Overall	Patients diagnosed with AD and mixed dementia	Patients without depression	Patients with baseline MMSE ≥ 10 and without depression
Non-use	Ref	Ref	Ref	Ref
Antidepressants ≤ 0.5 DDD	− 0.20 (− 0.33, − 0.08)	− 0.15 (− 0.30, 0.01)	− 0.28 (− 0.42, − 0.15)	− 0.28 (− 0.42, − 0.15)
Antidepressants > 0.5/ ≤ 1.0 DDD	− 0.33 (− 0.43, − 0.23)	− 0.33 (− 0.47, − 0.20)	− 0.29 (− 0.40, − 0.18)	− 0.28 (− 0.39, −0.16)
Antidepressants > 1.0 DDD	− 0.37 (− 0.48, − 0.27)	− 0.35 (− 0.49, − 0.21)	− 0.40 (− 0.52, − 0.28)	− 0.39 (− 0.51, − 0.27)
Non-use	Ref	Ref	Ref	Ref
SSRIs ≤ 0.5 DDD	− 0.32 (− 0.48, − 0.17)	− 0.30 (− 0.50, − 0.10)	− 0.48 (− 0.66, − 0.30)	− 0.48 (− 0.66, − 0.30)
SSRIs > 0.5/ ≤ 1.0 DDD	− 0.41 (− 0.53, − 0.29)	− 0.45 (− 0.60, − 0.30)	− 0.35 (− 0.48, − 0.22)	− 0.33 (− 0.46, − 0.20)
SSRIs > 1.0 DDD	− 0.42 (− 0.55, − 0.30)	− 0.36 (− 0.53, − 0.20)	− 0.43 (− 0.56, − 0.29)	− 0.41 (− 0.55, − 0.28)
Non-use	Ref	Ref	Ref	Ref
Others ≤ 0.5 DDD	− 0.04 (− 0.24, 0.16)	0.02 (− 0.23, 0.27)	− 0.08 (− 0.29, 0.13)	− 0.08 (− 0.29, 0.13)
Others > 0.5/ ≤ 1.0 DDD	− 0.25 (− 0.44, − 0.07)	− 0.33 (− 0.57, − 0.09)	− 0.29 (− 0.49, − 0.09)	− 0.29 (− 0.48, − 0.09)
Others > 1.0 DDD	− 0.44 (− 0.66, − 0.23)	− 0.58 (− 0.84, − 0.31)	− 0.52 (− 0.75, − 0.30)	− 0.52 (− 0.74, − 0.29)

*Abbreviations:* *SveDem* the Swedish Registry for Cognitive/Dementia Disorders, *CIs* Confidence intervals, *MMSE* Mini-Mental State Examination, *AD* Alzheimer’s disease, *Mixed* Mixed dementia, *SSRIs* Selective serotonin reuptake inhibitors, *Others* Other antidepressants

Adjused for age, sex, calendar year of diagnosis, the type of dementia, MMSE score at diagnosis, coresident status, care unit, depression, fracture, Charlson Comorbidity Index score, medications (angiotensin-converting enzyme inhibitors (ACEIs)/angiotensin receptor blockers (ARBs), β-blocking agents, calcium channel blockers, nonsteroidal anti-inflammatory drugs, diuretics, lipid-modifying agents, antiplatelets, antipsychotics, anxiolytics, and hypnotics). For patients diagnosed with AD and mixed dementia, models were further adjusted for cholinesterase inhibitors and memantine

### Subgroup analysis of antidepressant and cognitive decline by dementia subtypes

Subgroup analysis of the associations between antidepressants and cognitive decline is shown in Table 4. Compared with patients who were women, had higher MMSE score at baseline, using anxiolytics and hypnotics, the effect size of cognitive decline was greater in those who were men, had lower MMSE score, not using anxiolytics or hypnotics. Modifying effects of sex and use of anxiolytics on the association between antidepressant use and cognitive decline were observed (*P *_for interaction_ < 0.05). A slower cognitive decline was observed in younger (< 78 years) patients with FTD (*β* = 1.61 points/year; 95% CI, 0.05 to 3.17). Patients with the lowest initial MMSE scores (0–9) exhibited the greatest subsequent cognitive decline (*β* = − 1.51 points/year; 95% CI, − 2.82 to − 0.20). Furthermore, the results were similar between users and non-users of ChEIs; among patients not using memantine, compared with non-use, antidepressant use showed faster cognitive decline for patients diagnosed with AD and mixed dementia (*β* = − 0.30 points/year; 95% CI, − 0.45 to − 0.15), and for LBD (*β* = − 0.85 points/year; 95% CI, − 1.64 to − 0.06) (Additional file 1: Figure S4 and Additional file 2: Table S11). There were no significant differences between subgroups of coresident status, type of diagnostic unit, living in nursing home, calendar year of dementia diagnosis, and current use of antipsychotics on cognitive decline.

**Table 4:** Subgroup analysis of antidepressants and cognitive decline

Variables	All dementias (*n* = 18 740)	AD and mixed (*n* = 11 415)^a^	VaD (*n* = 2194)	LBD (*n* = 587)^a^	FTD (*n* = 201)	Other dementias (*n* = 4 343)
	*β* (95%CI)					
Sex						
Men	− 0.49 (− 0.63, − 0.36)^b,c^	− 0.53 (− 0.71, − 0.34)^b^	− 0.39 (− 0.74, − 0.04)^b^	− 0.36 (− 1.01, 0.29)	0.76 (− 1.18, 2.70)	− 0.44 (− 0.73, − 0.16)^b,c^
Women	− 0.14 (− 0.26, − 0.02)^b^	− 0.10 (− 0.26, 0.05)	− 0.13 (− 0.48, 0.23)	0.60 (− 0.52, 1.73)	1.84 (− 0.15, 3.83)	− 0.25 (− 0.49, − 0.01)^b^
Age at dementia diagnosis, y						
< 78 years	− 0.30 (− 0.43, − 0.17)^b^	− 0.21 (− 0.37, − 0.05)^b^	− 0.57 (− 0.99, − 0.15^b^	− 0.15 (− 0.88, 0.57)	1.61 (0.05, 3.17)^b^	− 0.55 (− 0.86, − 0.23)^b^
≥ 78 years	− 0.26 (− 0.39, − 0.13)^b^	− 0.30 (− 0.48, − 0.11)^b^	− 0.11 (− 0.46, 0.23)	− 0.10 (− 1.21, 1.00)	0.29 (− 2.50, 3.08)	− 0.21 (− 0.45, 0.02)
Coresident status						
Cohabiting	− 0.29 (− 0.41, − 0.17)^b^	− 0.27 (− 0.42, − 0.13)^b^	− 0.30 (− 0.65, 0.05)	− 0.37 (− 1.08, 0.34)	1.03 (− 0.65, 2.71)	− 0.28 (− 0.52, − 0.03)^b^
Living alone	− 0.30 (− 0.45, − 0.15)^b^	− 0.27 (− 0.48, − 0.06)^b^	− 0.21 (− 0.63, 0.21)	0.71 (− 0.38, 1.80)	1.59 (− 0.52, 3.70)	− 0.41 (− 0.69, − 0.12)^b^
Type of diagnostic unit						
Specialist care	− 0.23 (− 0.37, − 0.10)^b^	− 0.22 (− 0.37, − 0.07)^b^	− 0.58 (− 1.06, − 0.11)^b^	− 0.12 (− 0.77, 0.53)	1.33 (− 0.04, 2.69)	− 0.41 (− 0.92, 0.09)
Primary care	− 0.30 (− 0.42, − 0.17)^b^	− 0.33 (− 0.52, − 0.13)^b^	− 0.13 (− 0.43, 0.16)	− 0.26 (− 2.03, 1.50)	− 1.17 (− 4.02, 1.69)	− 0.32 (− 0.53, − 0.11)^b^
Nursing home						
No	− 0.30 (− 0.39, − 0.21)^b^	− 0.29 (− 0.41, − 0.17)^b^	− 0.26 (− 0.52, 0.00)^b^	0.00 (− 0.58 − 0.59)	1.33 (− 0.09, 2.75)	− 0.36 (− 0.57, − 0.16)^b^
Yes	− 0.27 (− 0.67, 0.13)	− 0.02 (− 0.65, 0.60)	− 0.37 (− 1.31, 0.58)	− 1.37 (− 3.75, 1.01)	NA	− 0.23 (− 0.90, 0.44)
Calendar year of diagnosis						
2007–2012	− 0.31 (− 0.44, − 0.19)^b^	− 0.25 (− 0.40, − 0.10)^b^	− 0.49 (− 0.83, − 0.14)^b^	− 0.26 (− 0.97, 0.45)	1.24 (− 0.41, 2.88)	− 0.32 (− 0.58, − 0.07)^b^
2013–2018	− 0.31 (− 0.47, − 0.15)^b^	− 0.26 (− 0.47, − 0.06)^b^	− 0.23 (− 0.64, 0.18)	− 0.03 (− 0.97, 0.91)	1.11 (− 1.28, 3.50)	− 0.45 (− 0.75, − 0.15)^b^
MMSE score at indexdate						
0–9	− 1.51 (− 2.82, − 0.20)^b^	− 2.42 (− 4.81, − 0.04)^b^	− 2.11 (− 5.96, 1.74)	NA	− 2.11 (− 5.96, 1.74)	− 0.48 (− 2.84, 1.87)
10–19	− 0.29 (− 0.53, − 0.06)^b^	− 0.37 (− 0.70, − 0.05)^b^	− 0.07 (− 0.68, 0.54)	0.27 (− 1.10, 1.64)	2.48 (− 0.69, 5.66)	− 0.15 (− 0.57, 0.27)
20–24	− 0.28 (− 0.41, − 0.14)^b^	− 0.21 (− 0.39, − 0.03)^b^	− 0.17 (− 0.55, 0.21)	− 0.37 (− 1.14, 0.40)	− 0.62 (− 2.21, 0.98)	− 0.41 (− 0.69, − 0.14)^b^
25–30	− 0.19 (− 0.34, − 0.05)^b^	− 0.17 (− 0.34, 0.01)	− 0.39 (− 0.82, 0.04)	0.23 (− 0.80, 1.26)	1.42 (− 0.72, 3.57)	− 0.25 (− 0.55, 0.05)
Antipsychotics						
No	− 0.31 (− 0.40, − 0.21)^b^	− 0.29 (− 0.41, − 0.17)^b^	− 0.22 (− 0.48, 0.03)	− 0.21 (− 0.83, 0.40)	1.43 (0.05, 2.80)^b^	− 0.37 (− 0.57, − 0.17)^b^
Yes	− 0.24 (− 0.89, 0.41)	0.01 (− 1.01, 1.03)	− 1.69 (− 3.00, − 0.38)^b^	0.36 (− 1.09, 1.80)	− 3.91 (− 15.63, 7.82)	− 0.43 (− 1.61, 0.74)
Anxiolytics						
No	− 0.36 (− 0.46, − 0.26)^b,c^	− 0.32 (− 0.45, − 0.19)^b^	− 0.32 (− 0.59, − 0.06)^b^	− 0.22 (− 0.83, 0.39)	1.33 (− 0.07, 2.72)	− 0.47 (− 0.69, − 0.25)^b,c^
Yes	− 0.12 (− 0.37, 0.12)	− 0.17 (− 0.49, 0.15)	− 0.32 (− 1.13, 0.50)	0.23 (− 1.23, 1.69)	− 1.08 (− 4.71, 2.56)	0.05 (− 0.39, 0.49)
Hypnotics						
No	− 0.36 (− 0.47, − 0.25)^b^	− 0.30 (− 0.44, − 0.17)^b^	− 0.38 (− 0.70, − 0.05)^b^	− 0.42 (− 1.10, 0.27)	1.21 (− 0.41, 2.84)	− 0.42 (− 0.66, − 0.19)^b^
Yes	− 0.23 (− 0.41, − 0.04)^b^	− 0.30 (− 0.55, − 0.06)^b^	− 0.16 (− 0.70, 0.39)	0.34 (− 0.75, 1.44)	1.05 (− 1.17, 3.27)	− 0.24 (− 0.60, 0.12)

Adjused for age, sex, calendar year of diagnosis, the type of dementia, Mini-Mental State Examination score at diagnosis, coresident status, care unit, depression, fracture, Charlson Comorbidity Index score, medications (angiotensin-converting enzyme inhibitors (ACEIs)/angiotensin receptor blockers (ARBs), β-blocking agents, calcium channel blockers, nonsteroidal anti-inflammatory drugs, diuretics, lipid-modifying agents, antiplatelets, antipsychotics, anxiolytics, and hypnotics)

*Abbreviations:* *SveDem* the Swedish Registry for Cognitive/Dementia Disorders, *AD* Alzheimer’s disease, *Mixed* Mixed dementia, *VaD* Vascular dementia, *LBD* Parkinson’s disease with dementia and dementia with Lewy bodies, *FTD* Frontotemporal dementia, *NA* Not available

^a^Further adjusted for ChEIs and memantine

^b^*P* < .05

^c^*P* for interaction of sex and anxiolytics with antidepressant < .05

### Antidepressant and severe dementia, fracture, and death

Compared with non-use, antidepressant use was associated with higher risk of all-cause mortality (HR = 1.07; 95% CI, 1.01 to 1.13) and fracture (HR = 1.18; 95% CI, 1.10 to 1.26) (Additional file 2: Table S12-S14). Higher dispensed dose (> 1.0 DDD) of SSRIs were associated with increased risk of severe dementia (HR = 1.35; 95% CI, 1.02 to 1.80), all-cause mortality (HR = 1.18; 95% CI, 1.07 to 1.31), and fracture (HR = 1.25; 95% CI, 1.10 to 1.43), compared with non-use (Additional file 2: Table S12-S14). Consistent results were observed in patients with AD and mixed dementia (Additional file 2: Table S15).

## Discussion

In this nationwide cohort study, we found that the current use of antidepressants was associated with faster cognitive decline in patients with dementia compared with non-use, and this association was driven mainly by patients with severe dementia. Higher dispensed doses of SSRIs were associated with more cognitive decline during follow-up, and higher risk of severe dementia, fracture, and all-cause mortality.

Previous studies of antidepressant use and cognitive decline in people with dementia have shown mixed findings. Most clinical trials reporting the effect of antidepressants on neuropsychiatric symptoms and cognition, did so as a secondary or safety outcome [43, 44]. In the majority of the cases reported, antidepressants had the same effect on cognition as placebo [44–47], but some reported a significant decline on the MMSE scores in antidepressant users [43, 48]. Many of the clinical trials were too small to provide precise estimates of the moderate benefits on cognition that might realistically be expected [49]. A recently conducted RCT reported worsening of cognition in patients with AD who used citalopram over 9 weeks [43]. In contrast, other RCTs in AD indicated that another two SSRIs, sertraline and escitalopram, had no effect on cognitive function [49, 50]. Individual antidepressants even within same class cannot be considered identical drugs. Evidence has shown some differences regarding the speed of onset of response and adverse events among individual SSRIs [51]. Recent findings demonstrated that specifically sertraline, escitalopram, and mirtazapine had relatively higher responses and lower dropout profile compared to other antidepressants [52]. These differences may influence medication choice for a given patient and may show varied effect on dementia progression. More studies are needed to explore the underlying mechanisms, optimal timing for intervention, and the types of patients who would benefit the most from these specific medications.

Our study showed significantly faster cognitive decline in patients with SSRIs (i.e., citalopram, sertraline, and escitalopram) compared to non-use. However, the magnitude of the effect of citalopram (0.41 points/year), sertraline (0.25 points/year), and escitalopram (0.76 points/year) appears to be lower than the minimum clinically significant change in MMSE score of 1–3 points [53]. A meta-analysis that included 15 RCTs involving a total of 1616 patients with AD found that second-generation antidepressants had no effect on global cognition measured by MMSE, and this remained in subgroup analyses of duration of medication, drug classes, combination with anti-dementia medication, various NPS, and degree of AD [47]. However, results from meta-analyses may be biased without adjustment for the clinical status of patients such as chronic physical disease and are not quantitatively assessed since most of the trials included used flexible doses of antidepressants. Sertraline and escitalopram are first hand choices for depression among older individuals in Sweden [8]. However, antidepressants do not seem to work as well in patients with dementia, possibly because “depression in dementia is a different illness” than depression in people with intact cognition [54]. Cognitive control dysfunction in dementia appears to decrease the effectiveness of some SSRIs [9]. In our study, we found SSRIs were associated with a small negative impact on cognitive functioning, but its clinical significance is uncertain.

Evidence regarding the long-term effect of antidepressants on cognitive decline in VaD is lacking. In previous open-label studies in patients with VaD [21], MMSE score increased significantly in the fluoxetine (SSRI) group but not in the control group. Another study conducted in patients with vascular cognitive impairment without dementia [55] found that, compared with controls, fluoxetine (SSRI) was associated with better performance on Ten Point Clock drawing test, indicating better cognitive functioning and greater ability to understand spatial relationships, plan, and execute tasks, but no significant differences in change of Alzheimer’s Disease Assessment Scale cognitive subscale score. In this study, we found that antidepressant use was associated with a significant decline in cognition in patients with VaD compared to non-use. Furthermore, we also observed a slower cognitive decline in younger (< 78 years) patients with FTD. In line with our observation, Laura et al. [56] found that citalopram can partially restore the dysfunctional prefrontal cortical systems by increasing serotonergic neurotransmission in FTD. Future long-term studies are warranted to validate our findings.

Some antidepressants, such as TCAs, are anticholinergic and combining these with acetylcholinesterase inhibitors is counterintuitive due to their conflicting mechanisms of action [57]. Our study showed the association between antidepressant use and cognitive decline was not modified by use of ChEIs but was worse in non-users of memantine. In a RCT (*n* = 95) among older adults with major depression and memory complains, a combination of memantine and escitalopram significantly improved delayed recall and executive functioning at 12 months [58]. However, the differences in specific antidepressants or anti-dementia drugs, sample size, and cognitive function at baseline might be the main reason related to the heterogeneity between studies.

Population-based cohort studies have found that antidepressant use was related to no or lower risks of all-cause mortality in patients with dementia [59, 60], but increased risk of fracture [61, 62]. In our study, we found that SSRIs were associated with increased risks of all-cause mortality and fracture compared with no current treatment. Thus, careful and regular monitoring is needed to assess the risks and benefits of different antidepressants and decrease the risk of adverse events.

This study has several strengths, including a large nationally representative cohort of individuals with dementia, long follow-up assessments, and a range of different types of dementia. In addition, demographic characteristics, medical disorders, and characteristics related to dementia were also explored. To our knowledge, this is the first systematic assessment of the long-term effects of commonly used antidepressants on cognition in patients with dementia. Our medication exposure was time-dependent, taking into account the change in prescribing patterns that occur after dementia diagnosis and thus more accurately reflective of medication use at the time of event. Furthermore, we had detailed information on prescriptions for antidepressants throughout the follow-up period, so we could do comprehensive analyses investigating effects of individual drugs and dose.

This study has limitations. The main concern is indication bias, which occurs when patients are prescribed drugs for a condition that is itself associated with the outcome of interest. Depression has been linked with dementia and cognitive impairment in normal people with depression [41, 42], which means that the associations with antidepressants may be due to depression for which it was prescribed rather than to the drug itself. To reduce this bias, we repeated and restricted our main analysis to include only patients without depression and found consistent results. In addition, in this study only patients who were new users of antidepressants were included, previous users of antidepressants with depression were not included. However, the clinical diagnosis of depression was obtained through specialized in- and out-patient care, which primarily represents more severe manifestations: less severe depression is generally managed through primary care and would have been missed. And there is an overlap in symptoms between depression and dementia, making diagnosing depression particularly challenging in these cases. Furthermore, we did not have information on the severity of depression and actual intake of antidepressants, only dispensation. Residual confounding due to lack of diagnosis of depression in dementia would thus reduce the differences between antidepressants users and non-users since more individuals are likely to end up erroneously in the group of non-users. However, many patients with dementia seem to have received antidepressant medications for other neuropsychiatric symptoms, as the sensitivity analyses among non-depressed patients did not change the results. But the direct comparisons among treated groups exhibit reduced susceptibility to confounding by indication or factors influencing treatment prescription. Second, although direct comparisons between antidepressant classes or drugs could to some extent reduce the influence of neuropsychiatric symptoms, they may still be influenced by channeling bias. This bias can occur when distinct antidepressant drugs with similar indications are prescribed based on varying patient characteristics [52]. Third, the severity of dementia could independently contribute to cognitive decline, making it difficult to definitively attribute the observed effects solely to antidepressant use. However, we observed antidepressant use was associated with faster cognitive decline in patients with different MMSE scores at dementia diagnosis, though the effect size of cognitive decline was greater in patients with lower MMSE score. Future research is needed to further elucidate the complex interplay between antidepressant use, dementia severity, and cognitive decline. Fourth, SveDem is a real-world database, which suffers from significant patient loss to follow-up. In this study, we calculated and adjusted the inverse probability of censoring weighting to address the issue of selective dropouts. Furthermore, the national coverage of SveDem for new dementia cases is not absolute, it covers 100% of memory clinics and 75% of primary care units, and almost one-third of all expected new dementia cases in Sweden [63, 64]. Thus, the generalizability of our findings to other populations remains to be studied. However, the dementia diagnostic workup follows standard clinical practice, and few patients have a changed dementia diagnosis at follow-up, which suggests adequate diagnostic accuracy [65]. Lastly, some of the analyses were imprecisely estimated due to the small number of patients with some individual antidepressants and in stratified analysis.

## Conclusions

In this cohort study, use of antidepressants was associated with faster cognitive decline in patients with dementia, in particular SSRIs (i.e., citalopram, sertraline and escitalopram) and mirtazapine. These effects appeared to be more pronounced in patients with more severe dementia. Compared with sertraline, escitalopram presented faster cognitive decline, while citalopram was linked to a slower cognitive decline. Additionally, higher dispensed doses of SSRIs were associated with greater cognitive decline, as well as increased risks of severe dementia, all-cause mortality, and fracture. Our study cannot distinguish whether these findings are due to the antidepressants or the underlying psychiatric indication.

## Supplementary Information

Additional file 1: Figure S1. Flowchart of study patients with dementia. Figure S2. Distribution of dosages of antidepressants by antidepressant class. Figure S3. Estimated MMSE trajectories between use of antidepressants and non-use by dose in patients from SveDem, 2007–2018 a . Figure S4. Estimated MMSE trajectories between use of antidepressants and non-use stratified by dementia medications in patients from SveDem, 2007–2018^a^.

Additional file 2: Table S1. ICD-10 codes for dementia and subtypes. Table S2. ATC codes for antidepressants. Table S3. ICD-10 codes for comorbidities. Table S4. ATC codes for medications. Table S5. Defined daily doses (DDD) by antidepressant classes. Values are numbers of prescriptions (column percentages). Table S6. Baseline characteristics of dementia patients. Table S7. Associations of antidepressants dose with cognitive decline by antidepressant class in patients from SveDem, 2007–2018. Table S8. Associations of antidepressants with cognitive decline by antidepressant class in patients diagnosed with AD and mixed dementia from SveDem. Table S9. Associations between antidepressants and cognitive decline in patients without depression, 2007–2018. Table S10. Associations between antidepressants and cognitive decline in patients with baseline MMSE ≥ 10 and without depression, 2007–2018. Table S11. Stratified analysis of associations between antidepressants and cognitive decline by dementia medications in patients with AD and mixed dementia and LBD from SveDem, 2007–2018. Table S12. Hazard ratios for adverse outcomes by antidepressant class and dose in patients from SveDem, 2007–2018. Table S13. Incidence Rate for adverse outcomes by antidepressant class in patients from SveDem, 2007–2018. Table S14. Incidence rate for adverse outcomes by antidepressant class and dose in patients from SveDem, 2007–2018. Table S15. Incidence Rate and hazard ratios for adverse outcomes by antidepressant class and dementia subtypes in patients from SveDem, 2007–2018.

## Data Availability

Following the Swedish and EU legislation, the data are not available for public access. In order to obtain the data from Swedish registries, researches must apply to the steering committees of the registries as well as relevant government authorities, after obtaining the ethical approval.

## Abbreviations

AD: Alzheimer’s dementia
CCIs: Charlson Comorbidity Index score
ChEIs: Cholinesterase inhibitors
CIs: Confidence intervals
DDD: Defined daily doses
DLB: Dementias with Lewy bodies
FTD: Frontotemporal dementia
HRs: Hazard ratios
ICD-10: International Classification of Diseases, Tenth Revision
MCI: Mild cognitive impairment
MMSE: Mini-Mental State Examination
PDD: Parkinson’s disease dementia
RCTs: Randomized controlled trials
SNRIs: Serotonin norepinephrine reuptake inhibitors
SSRIs: Selective serotonin reuptake inhibitors
SveDem: Swedish Registry for Cognitive/Dementia Disorders
TCAs: Tricyclic antidepressants
VaD: Vascular dementia
