# Supplementary material for: Antidepressant use and cognitive decline in patients with dementia: a national cohort study
Source: BMC Med. 2025 Feb 25;23:82. doi: 10.1186/s12916-025-03851-3 (PMC11854023; doi:10.1186/s12916-025-03851-3)
Supplement: Supplementary file 1 — Additional file 1: Figure S1. Flowchart of study patients with dementia. Figure S2. Distribution of dosages of antidepressants by antidepressant class. Figure S3. Estimated MMSE trajectories between use of antidepressants and non-use by dose in patients from SveDem, 2007–2018 a . Figure S4. Estimated MMSE trajectories between use of antidepressants and non-use stratified by dementia medications in patients from SveDem, 2007–2018a. [file 12916_2025_3851_MOESM1_ESM.docx]

**Additional file 1:** **Figures S1-S4**

Figure S1. Flowchart of study patients with dementia

Figure S2. Distribution of dosages of antidepressants by antidepressant class

Figure S3. Estimated MMSE trajectories between use of antidepressants and non-use by dose in patients from SveDem, 2007-2018 ^a^

Figure S4. Estimated MMSE trajectories between use of antidepressants and non-use stratified by dementia medications in patients from SveDem, 2007-2018 ^a^


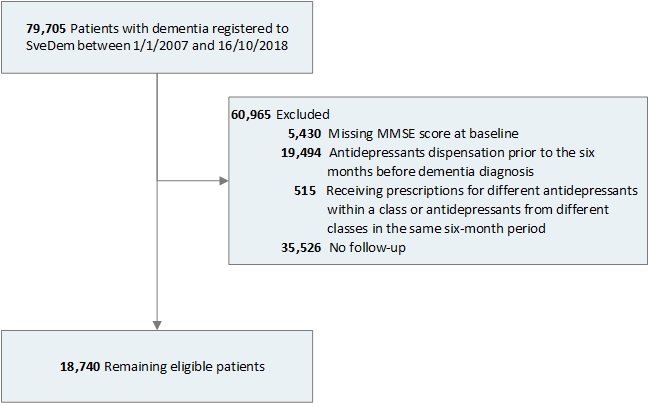


Figure S1. Flowchart of study patients with dementia

Abbreviations: SveDem, the Swedish Registry for Cognitive/Dementia Disorders; MMSE, Mini-Mental State Examination.


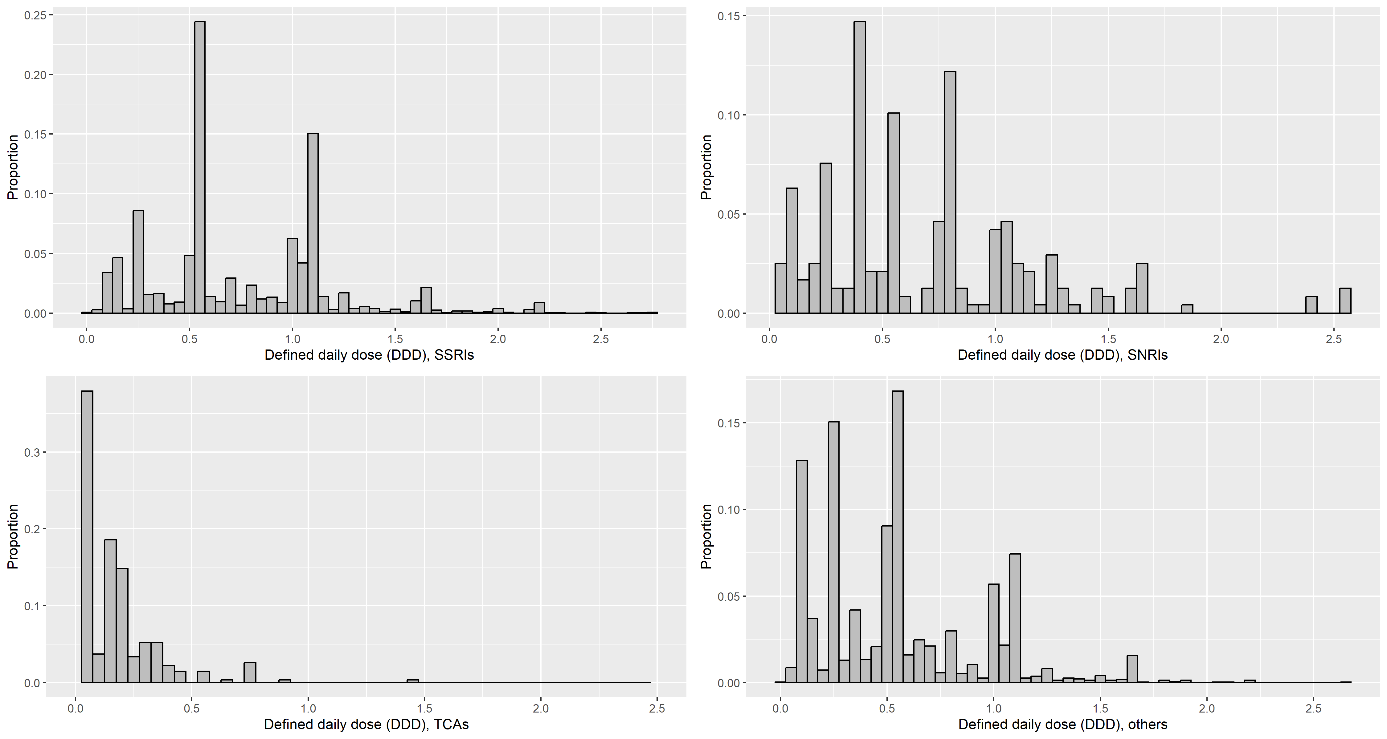


Figure S2. Distribution of dosages of antidepressants by antidepressant class

Abbreviations: SSRIs, selective serotonin reuptake inhibitors; SNRIs, serotonin and norepinephrine reuptake inhibitors; TCAs, Tricyclic antidepressants; Others, other antidepressants.

**A B C D**
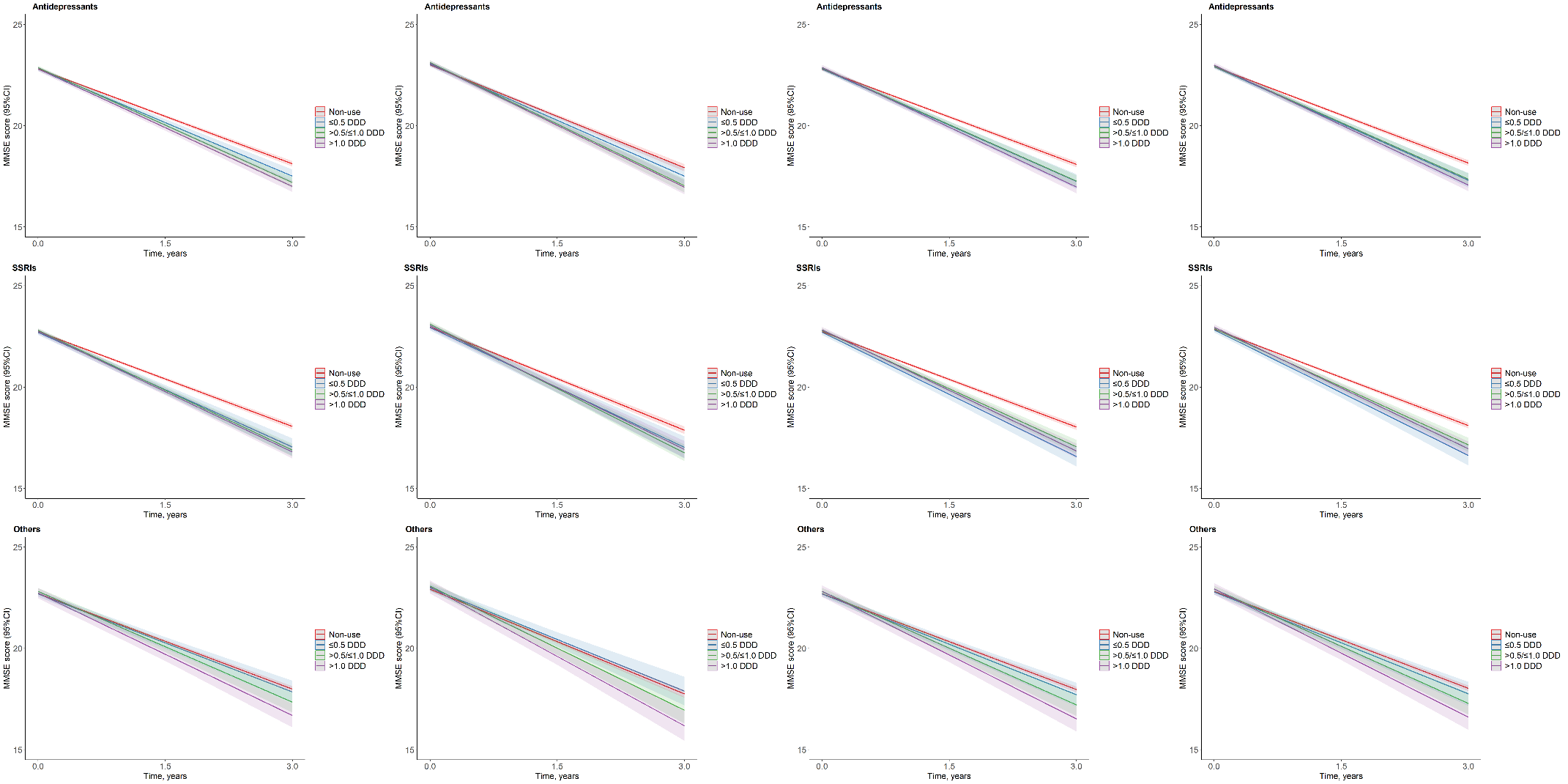


Figure S3. Estimated MMSE trajectories between use of antidepressants and non-use by dose in patients from SveDem, 2007-2018 ^a^

A. Among all patients, B. Among patients diagnosed with AD and mixed dementia, C. Among patients without depression, D. Among patients with baseline MMSE ≥10 and without depression.

Abbreviations: SveDem, the Swedish Registry for Cognitive/Dementia Disorders; CIs, confidence intervals; MMSE, Mini-Mental State Examination; AD, Alzheimer’s disease; Mixed, mixed dementia; SSRIs, selective serotonin reuptake inhibitors; Others, other antidepressants. ^a^, Estimated MMSE trajectories from mixed model, adjusted for age, sex, calendar year of diagnosis, the type of dementia, MMSE score at diagnosis, coresident status, care unit, depression, fracture, Charlson Comorbidity Index score, medications (angiotensin-converting enzyme inhibitors (ACEIs)/angiotensin receptor blockers (ARBs), β-blocking agents, calcium channel blockers, nonsteroidal anti-inflammatory drugs, diuretics, lipid modifying agents, antiplatelets, antipsychotics, anxiolytics and hypnotics). For patients diagnosed with AD and mixed dementia and LBD, models further adjusted for cholinesterase inhibitors and memantine.

**B. Among patients with LBD**


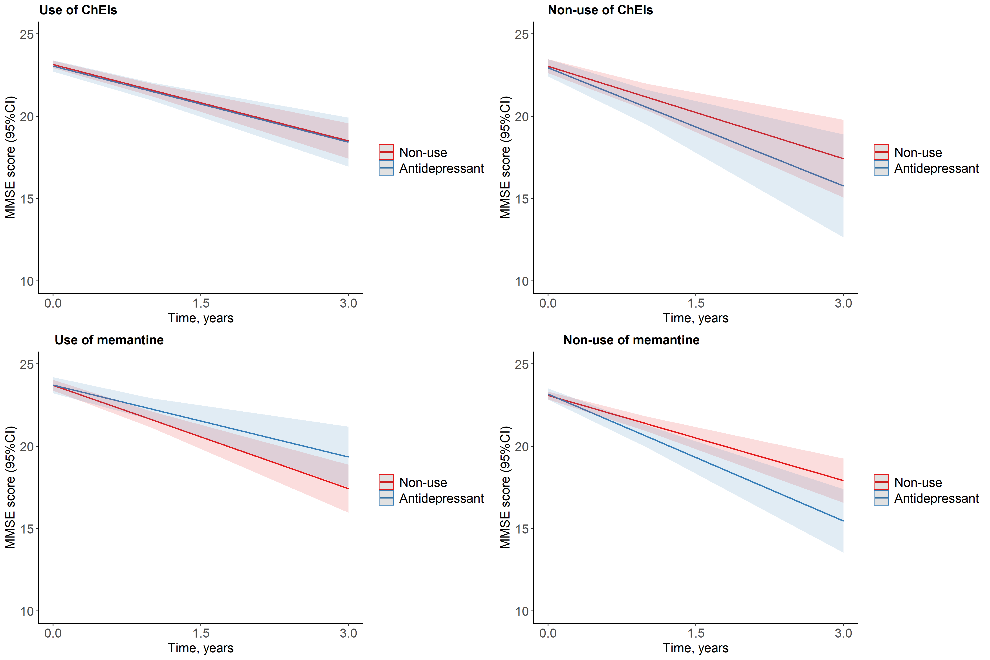


**A. Among patients with AD and mixed dementia**


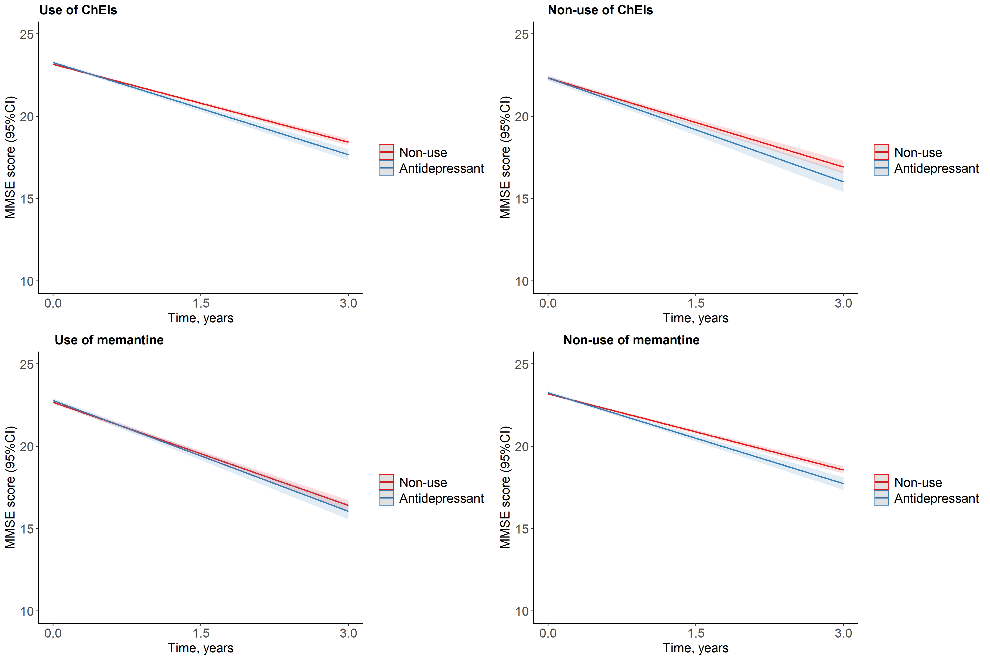


Figure S4. Estimated MMSE trajectories between use of antidepressants and non-use stratified by dementia medications in patients from SveDem, 2007-2018 ^a^

Abbreviations: SveDem, the Swedish Registry for Cognitive/Dementia Disorders; CIs, confidence intervals; MMSE, Mini-Mental State Examination; AD, Alzheimer’s disease; Mixed, mixed dementia; LBD, Parkinson’s disease with dementia and dementia with Lewy bodies. ^a^, Estimated MMSE trajectories from mixed model, adjusted for age, sex, calendar year of diagnsis, the type of dementia, MMSE score at diagnosis, coresident status, care unit, depression, fracture, Charlson Comorbidity Index score, medications (angiotensin-converting enzyme inhibitors (ACEIs)/angiotensin receptor blockers (ARBs), β-blocking agents, calcium channel blockers, nonsteroidal anti-inflammatory drugs, diuretics, lipid modifying agents, antiplatelets, antipsychotics, anxiolytics and hypnotics, cholinesterase inhibitors and memantine).
