# Supplementary material for: Antidepressant use and cognitive decline in patients with dementia: a national cohort study
Source: BMC Med. 2025 Feb 25;23:82. doi: 10.1186/s12916-025-03851-3 (PMC11854023; doi:10.1186/s12916-025-03851-3)
Supplement: Supplementary file 2 — Additional file 2: Table S1. ICD-10 codes for dementia and subtypes. Table S2. ATC codes for antidepressants. Table S3. ICD-10 codes for comorbidities. Table S4. ATC codes for medications. Table S5. Defined daily doses (DDD) by antidepressant classes. Values are numbers of prescriptions (column percentages). Table S6. Baseline characteristics of dementia patients. Table S7. Associations of antidepressants dose with cognitive decline by antidepressant class in patients from SveDem, 2007–2018. Table S8. Associations of antidepressants with cognitive decline by antidepressant class in patients diagnosed with AD and mixed dementia from SveDem. Table S9. Associations between antidepressants and cognitive decline in patients without depression, 2007–2018. Table S10. Associations between antidepressants and cognitive decline in patients with baseline MMSE ≥ 10 and without depression, 2007–2018. Table S11. Stratified analysis of associations between antidepressants and cognitive decline by dementia medications in patients with AD and mixed dementia and LBD from SveDem, 2007–2018. Table S12. Hazard ratios for adverse outcomes by antidepressant class and dose in patients from SveDem, 2007–2018. Table S13. Incidence Rate for adverse outcomes by antidepressant class in patients from SveDem, 2007–2018. Table S14. Incidence rate for adverse outcomes by antidepressant class and dose in patients from SveDem, 2007–2018. Table S15. Incidence Rate and hazard ratios for adverse outcomes by antidepressant class and dementia subtypes in patients from SveDem, 2007–2018. [file 12916_2025_3851_MOESM2_ESM.docx]

**Additional file 2: Table S1-S15**

Table S1. ICD-10 codes for dementia and subtypes

Table S2. ATC codes for antidepressants

Table S3. ICD-10 codes for comorbidities

Table S4. ATC codes for medications

Table S5. Defined daily doses (DDD) by antidepressant classes. Values are numbers of prescriptions (column percentages)

Table S6. Baseline characteristics of dementia patients

Table S7. Associations of antidepressants dose with cognitive decline by antidepressant class in patients from SveDem, 2007-2018

Table S8. Associations of antidepressants with cognitive decline by antidepressant class in patients diagnosed with AD and mixed dementia from SveDem

Table S9. Associations between antidepressants and cognitive decline in patients without depression, 2007-2018

Table S10. Associations between antidepressants and cognitive decline in patients with baseline MMSE ≥10 and without depression, 2007-2018

Table S11. Stratified analysis of associations between antidepressants and cognitive decline by dementia medications in patients with AD and mixed dementia and LBD from SveDem, 2007-2018

Table S12. Hazard ratios for adverse outcomes by antidepressant class and dose in patients from SveDem, 2007-2018

Table S13. Incidence Rate for adverse outcomes by antidepressant class in patients from SveDem, 2007-2018

Table S14. Incidence rate for adverse outcomes by antidepressant class and dose in patients from SveDem, 2007-2018

Table S15. Incidence Rate and hazard ratios for adverse outcomes by antidepressant class and dementia subtypes in patients from SveDem, 2007-2018

| Table S1. ICD-10 codes for dementia and subtypes | |
| --- | --- |
| Dementia subtypes | ICD-10 code |
| Dementia in Alzheimer’s disease | F00.0, F00.1, F00.9, G30.0, G30.1, G30.9 |
| Mixed dementia in Alzheimer’s disease and vascular dementia | F00.2, G30.8 |
| Vascular dementia (incl Subcortical vascular dementia) | F01.0, F01.1, F01.2, F01.3, F01.8, F01.9 |
| Lewy body dementia | F02.8, G31.8 |
| Frontotemporal dementia | F02.0, G31.0 |
| Dementia in Parkinson’s disease | F02.3, G20 |
| Unspecified dementia | F03 |
| Other dementia (dementia diagnosis expect for the above) | F02.1, F02.2, F02.4, F02.8 (excluding Lewy body dementia), F10.7A |
| Abbreviations: ICD-10, International Statistical Classification of Diseases and Related Health Problems, tenth revision. | |

| Table S2. ATC codes for antidepressants | |
| --- | --- |
| Medications | ATC code |
| Antidepressants | N06A |
| Selective serotonin reuptake inhibitors | N06AB |
| Fluoxetine | N06AB03 |
| Citalopram | N06AB04 |
| Paroxetine | N06AB05 |
| Sertraline | N06AB06 |
| Fluvoxamine | N06AB08 |
| Escitalopram | N06AB10 |
| Serotonin and norepinephrine reuptake inhibitors |  |
| Venlafaxine | N06AX16 |
| Duloxetine | N06AX21 |
| Tricyclic antidepressants | N06AA |
| Imipramine | N06AA02 |
| Clomipramine | N06AA04 |
| Trimipramine | N06AA06 |
| lofepramine | N06AA07 |
| Amitriptyline | N06AA09 |
| Nortriptyline | N06AA10 |
| Maprotiline | N06AA21 |
| Monoamine oxidase inhibitors | N06AF, N06AG |
| Phenelzine | N06AF03 |
| Tranylcypromine | N06AF04 |
| Moclobemide | N06AG02 |
| Other antidepressants | N06AX |
| Tryptophan | N06AX02 |
| Mianserin | N06AX03 |
| Trazodone | N06AX05 |
| Nefazodone | N06AX06 |
| Mirtazapine | N06AX11 |
| Bupropion | N06AX12 |
| Tianeptine | N06AX14 |
| Reboxetine | N06AX18 |
| Agomelatine | N06AX22 |
| Vortioxetine | N06AX26 |
| Abbreviations: ATC, Anatomical Therapeutic Chemical. | |

| Table S3. ICD-10 codes for comorbidities | |
| --- | --- |
| Comorbidities | ICD-10 code |
| Atrial fibrillation | I48 |
| Cancer | C00 – C97 |
| Cerebrovascular diseases | G45, I60, I61, I62, I63, I64, I67, I69 |
| Congestive heart failure | I110, I130, I132, I255, I420, I426, I427, I428, I429, I43, I50 |
| Chronic obstructive pulmonary disease | J43, J44 |
| Diabetes | E100 – E107, E110 – E117, E120 – E127, E130 -E137, E140 – E147 |
| Hypertensive diseases | I10, I11, I12, I13, I14, I15, I16 |
| Liver diseases | B15, B16, B17, B18, B19, K754, K746, K73, K703, K709, I850, I859, I982, I983 |
| Myocardial infarction | I21, I22, I252 |
| Peripheral vascular diseases | I70, I71, I731, I738, I739, I771, I790, I792, K55 |
| Renal diseases | I120, I131, Z992, Z940, Z49, Q614, Q613, Q612, Q611, N250, N19, N18, N11, N057, N056, N055, N054, N053, N052, N037, N036, N035, N034, N033, N032, Z992 |
| Rheumatic diseases | M05, M06, M070, M071, M072, M073, M08, M123, M13, M30, M313, M314, M315, M316, M32, M33, M34, M350, M351, M353, M45, M46 |
| Depression | F32, F33, F34.1, F34.8, F34.9, F38, F39 |
| Fracture | S12, S22, S32, S42, S52, S62, S72, S82, S92 |
| Abbreviations: ICD-10, International Statistical Classification of Diseases and Related Health Problems, tenth revision. | |

| Table S4. ATC codes for medications | |
| --- | --- |
| Medications | ATC code |
| Angiotensin-converting enzyme inhibitors (ACEIs)/angiotensin receptor blockers (ARBs) | C09 |
| β-blocking agents | C07 |
| Calcium channel blockers | C08 |
| Diuretics | C03 |
| Nonsteroidal anti-inflammatory drugs | M01A |
| Lipid modifying agents | C10 |
| Antiplatelets | B01AC |
| Antipsychotics | N05A |
| Anxiolytics | N05B |
| Hypnotics/sedatives | N05C |
| Memantine | N06DX01 |
| Cholinesterase inhibitors | N06DA |
| Abbreviations: ATC, Anatomical Therapeutic Chemical. | |

| Table S5. Defined daily doses (DDD) by antidepressant classes. Values are numbers of prescriptions (column percentages) | | | | |
| --- | --- | --- | --- | --- |
| Defined daily dose (DDD) | Antidepressant classes | | | |
|  | SSRIs | SNRIs | TCAs | Others |
| ≤0.5 DDD | 2027 (26.3) | 98 (41.2) | 255 (94.8) | 1738 (47.1) |
| >0.5/≤1.0 DDD | 3266 (42.3) | 86 (36.1) | 13 (4.8) | 1394 (37.8) |
| >1.0 DDD | 2424 (31.4) | 54 (22.7) | 1 (0.4) | 556 (15.1) |
| Total | 7717 | 238 | 269 | 3688 |
| Abbreviations: SSRIs, selective serotonin reuptake inhibitors; SNRIs, serotonin and norepinephrine reuptake inhibitors; TCAs, Tricyclic antidepressants; Others, other antidepressants. | | | | |

| Table S6. Baseline characteristics of dementia patients | | | | |
| --- | --- | --- | --- | --- |
| Variables | Study cohort  (n=18 740) | Non-use  (n=14 469) | Antidepressants use (n=4 271) ^a^ | *P* |
| Age at dementia diagnosis, y | |  |  | <.001 |
| Mean (SD) | 78.2 (7.4) | 78.5 (7.4) | 77.1 (7.6) |  |
| < 78 | 7981 (42.6) | 5895 (40.7) | 2086 (48.8) |  |
| ≥ 78 | 10759 (57.4) | 8574 (59.3) | 2185 (51.2) |  |
| Sex |  |  |  | <.001 |
| Male | 8535 (45.5) | 6755 (46.7) | 1780 (41.7) |  |
| Female | 10205 (54.5) | 7714 (53.3) | 2491 (58.3) |  |
| Dementia types |  |  |  | <.001 |
| AD | 7727 (41.2) | 5951 (41.1) | 1776 (41.6) |  |
| Mixed | 3688 (19.7) | 2907 (20.1) | 781 (18.3) |  |
| VaD | 2194 (11.7) | 1666 (11.5) | 528 (12.4) |  |
| FTD | 201 (1.1) | 132 (0.9) | 69 (1.6) |  |
| LBD | 587 (3.1) | 403 (2.8) | 184 (4.3) |  |
| Other dementias | 4343 (23.2) | 3410 (23.6) | 933 (21.9) |  |
| MMSE score |  |  |  | <.001 |
| Mean (SD) | 22.1 (4.3) | 22.0 (4.3) | 22.5 (4.2) |  |
| 0-9 | 161 (0.9) | 131 (0.9) | 30 (0.7) |  |
| 10-19 | 4460 (23.8) | 3574 (24.7) | 886 (20.7) |  |
| 20-24 | 8159 (43.5) | 6347 (43.9) | 1812 (42.4) |  |
| 25-30 | 5960 (31.8) | 4417 (30.5) | 1543 (36.1) |  |
| Coresident status |  |  |  | 0.146 |
| Cohabiting | 11083 (59.1) | 8516 (58.9) | 2567 (60.1) |  |
| Living alone | 7657 (40.9) | 5953 (41.1) | 1704 (39.9) |  |
| Type of diagnostic unit |  |  |  | <.001 |
| Specialist care | 9824 (52.4) | 7398 (51.1) | 2426 (56.8) |  |
| Primary care | 8916 (47.6) | 7071 (48.9) | 1845 (43.2) |  |
| Calendar year of diagnosis | |  |  | <.001 |
| 2007-2009 | 2194 (11.7) | 1591 (11.0) | 603 (14.1) |  |
| 2010-2012 | 5859 (31.3) | 4397 (30.4) | 1462 (34.2) |  |
| 2013-2015 | 7105 (37.9) | 5540 (38.3) | 1565 (36.6) |  |
| 2016-2018 | 3582 (19.1) | 2941 (20.3) | 641 (15.0) |  |
| Comorbidities |  |  |  |  |
| Depression | 257 (1.4) | 55 (0.4) | 202 (4.7) | <.001 |
| Fracture | 1817 (9.7) | 1413 (9.8) | 404 (9.5) | 0.552 |
| Charlson Comorbidity Index score | |  |  | 0.826 |
| 0 | 12369 (66.0) | 9543 (66.0) | 2826 (66.2) |  |
| 1 | 2909 (15.5) | 2255 (15.6) | 654 (15.3) |  |
| 2 | 1936 (10.3) | 1504 (10.4) | 432 (10.1) |  |
| ≥3 | 1526 (8.1) | 1167 (8.1) | 359 (8.4) |  |
| Medication use |  |  |  |  |
| Angiotensin-converting enzyme inhibitors (ACEIs)/angiotensin receptor blockers (ARBs) | 8134 (43.4) | 6357 (43.9) | 1777 (41.6) | 0.007 |
| β-blocking agents | 7526 (40.2) | 5893 (40.7) | 1633 (38.2) | 0.004 |
| Calcium channel blockers | 5227 (27.9) | 4080 (28.2) | 1147 (26.9) | 0.086 |
| Diuretics | 7136 (38.1) | 5489 (37.9) | 1647 (38.6) | 0.459 |
| Lipid modifying agents | 7526 (40.2) | 5855 (40.5) | 1671 (39.1) | 0.116 |
| Nonsteroidal anti-inflammatory drugs | 4853 (25.9) | 3605 (24.9) | 1248 (29.2) | <.001 |
| Antiplatelets | 8044 (42.9) | 6216 (43.0) | 1828 (42.8) | 0.852 |
| Anxiolytics | 2567 (13.7) | 1621 (11.2) | 946 (22.2) | <.001 |
| Hypnotics | 4333 (23.1) | 3014 (20.8) | 1319 (30.9) | <.001 |
| Antipsychotics | 637 (3.4) | 431 (3.0) | 206 (4.8) | <.001 |
| Cholinesterase inhibitors ^b^ | 9055 (75.5) | 7013 (75.7) | 2042 (74.5) | 0.189 |
| Memantine ^b^ | 3691 (30.8) | 2723 (29.4) | 968 (35.3) | <.001 |
| Abbreviations: AD, Alzheimer’s disease; Mixed, mixed dementia; VaD, vascular dementia; LBD, Parkinson’s disease with dementia and dementia with Lewy bodies; FTD, frontotemporal dementia; SD, standard deviation; MMSE, Mini-Mental State Examination. | | | | |
| ^a^, received at least one prescription for an antidepressant during follow-up.  ^b^, among patients diagnosed with AD, mixed dementia and LBD. | | | | |

| Table S7. Associations of antidepressants dose (continuous) with cognitive decline (change in cognition slope, per y) by antidepressant class in patients from SveDem, 2007-2018 | | | | |
| --- | --- | --- | --- | --- |
| Antidepressant class | β (95%CIs) | | | |
|  | Overall | Patients diagnosed with AD and mixed dementia | Patients without depression | Patients with baseline MMSE ≥10 and without depression |
| Antidepressants | -0.20 (-0.27, -0.13) | -0.09 (-0.18, 0.00) | -0.20 (-0.28, -0.13) | -0.19 (-0.27, -0.12) |
| SSRIs | -0.22 (-0.29, -0.14) | -0.07 (-0.17, 0.03) | -0.22 (-0.30, -0.13) | -0.20 (-0.29, -0.12) |
| SNRIs | 0.05 (-0.50, 0.61) | 0.02 (-0.78, 0.82) | 0.11 (-0.50, 0.72) | 0.11 (-0.49, 0.72) |
| TCAs | 1.17 (-0.65, 2.98) | 1.83 (-0.23, 3.90) | 1.30 (-0.53, 3.13) | 1.34 (-0.49, 3.16) |
| Others | -0.20 (-0.36, -0.04) | -0.23 (-0.42, -0.04) | -0.24 (-0.41, -0.07) | -0.24 (-0.41, -0.07) |
| Abbreviations: CIs, confidence intervals; SveDem, the Swedish Registry for Cognitive/Dementia Disorders; SSRIs, selective serotonin reuptake inhibitors; Others, other antidepressants. | | | | |
| Adjused for age, sex, calendar year of diagnosis, the type of dementia, Mini-Mental State Examination score at diagnosis, coresident status, care unit, depression, fracture, Charlson Comorbidity Index score, medications (angiotensin-converting enzyme inhibitors (ACEIs)/angiotensin receptor blockers (ARBs), β-blocking agents, calcium channel blockers, nonsteroidal anti-inflammatory drugs, diuretics, lipid modifying agents, antiplatelets, antipsychotics, anxiolytics and hypnotics). For patients diagnosed with AD and mixed dementia, models were further adjusted for cholinesterase inhibitors and memantine. | | | | |

| Table S8. Associations of antidepressants with cognitive decline (change in cognition slope, per y) by antidepressant class in patients diagnosed with AD and mixed dementia from SveDem | | |
| --- | --- | --- |
| Antidepressant class | Patients diagnosed with AD and mixed dementia | |
|  | β (95%CIs) | *P* |
| Non-use | Ref |  |
| SSRIs | -0.39 (-0.53, -0.24) | <.001 |
| Others | -0.23 (-0.43, -0.03) | 0.028 |
|  |  |  |
| Non-use | Ref |  |
| Citalopram (SSRI) | -0.34 (-0.52, -0.16) | <.001 |
| Sertraline (SSRI) | -0.32 (-0.57, -0.09) | 0.008 |
| Escitalopram (SSRI) | -0.87 (-1.29, -0.46) | <.001 |
| Mirtazapine (Other) | -0.24 (-0.44, -0.03) | 0.023 |
|  |  |  |
| SSRIs | Ref |  |
| Others | 0.15 (-0.06, 0.37) | 0.168 |
|  |  |  |
| Sertraline (SSRI) | Ref |  |
| Citalopram (SSRI) | -0.04 (-0.31, 0.23) | 0.768 |
| Escitalopram (SSRI) | -0.53 (-1.01, -0.04) | 0.035 |
| Mirtazapine (Other) | 0.08 (-0.21, 0.37) | 0.603 |
| Abbreviations: SveDem, the Swedish Registry for Cognitive/Dementia Disorders; CIs, confidence intervals; AD, Alzheimer’s disease; mixed, mixed dementia; SSRIs, selective serotonin reuptake inhibitors; Others, other antidepressants. | | |
| Adjused for age, sex, calendar year of diagnosis, the type of dementia, Mini-Mental State Examination score at diagnosis, coresident status, care unit, depression, fracture, Charlson Comorbidity Index score, medications (angiotensin-converting enzyme inhibitors (ACEIs)/angiotensin receptor blockers (ARBs), β-blocking agents, calcium channel blockers, nonsteroidal anti-inflammatory drugs, diuretics, lipid modifying agents, antiplatelets, antipsychotics, anxiolytics, hypnotics, cholinesterase inhibitors and memantine). | | |

| Table S9. Associations between antidepressants and cognitive decline (change in cognition slope, per y) in patients without depression, 2007-2018 | | |
| --- | --- | --- |
| Dementia disorders | Patients without depression | |
|  | β (95%CIs) | *P* |
| Non-use | Ref |  |
| Antidepressants | -0.32 (-0.41, -0.22) | <.001 |
|  |  |  |
| Non-use | Ref |  |
| SSRIs | -0.40 (-0.52, -0.28) | <.001 |
| SNRIs | 0.27 (-0.33, 0.87) | 0.377 |
| TCAs | 0.22 (-0.31, 0.75) | 0.412 |
| Others | -0.24 (-0.40, -0.08) | 0.004 |
|  |  |  |
| Non-use | Ref |  |
| Citalopram (SSRI) | -0.42 (-0.56, -0.27) | <.001 |
| Sertraline (SSRI) | -0.28 (-0.49, -0.08) | 0.006 |
| Escitalopram (SSRI) | -0.74 (-1.10, -0.38) | <.001 |
| Venlafaxine (SNRI) | -0.01 (-0.74, 0.75) | 0.989 |
| Amitriptyline (TCA) | 0.22 (-0.31, 0.75) | 0.412 |
| Mirtazapine (Other) | -0.24 (-0.40, -0.08) | 0.004 |
|  |  |  |
| SSRIs | Ref |  |
| Others | 0.16 (-0.01, 0.34) | 0.064 |
|  |  |  |
| Sertraline (SSRI) | Ref |  |
| Citalopram (SSRI) | 0.28 (0.17, 0.39) | <.001 |
| Escitalopram (SSRI) | -0.42 (-84, 0.03) | 0.051 |
| Mirtazapine (Other) | 0.05 (-0.18, 0.28) | 0.656 |
| Abbreviations: CIs, confidence intervals; SSRIs, selective serotonin reuptake inhibitors; SNRIs, serotonin and norepinephrine reuptake inhibitors; TCAs, Tricyclic antidepressants; Others, other antidepressants. | | |
| Adjused for age, sex, calendar year of diagnosis, the type of dementia, Mini-Mental State Examination score at diagnosis, coresident status, care unit, depression, fracture, Charlson Comorbidity Index score, medications (angiotensin-converting enzyme inhibitors (ACEIs)/angiotensin receptor blockers (ARBs), β-blocking agents, calcium channel blockers, nonsteroidal anti-inflammatory drugs, diuretics, lipid modifying agents, antiplatelets, antipsychotics, anxiolytics and hypnotics). | | |

| Table S10. Associations between antidepressants and cognitive decline (change in cognition slope, per y) in patients with baseline MMSE ≥10 and without depression, 2007-2018 | | |
| --- | --- | --- |
| Dementia disorders | Patients with baseline MMSE ≥10 and without depression | |
|  | β (95%CIs) | *P* |
| Non-use | Ref |  |
| Antidepressants | -0.31 (-0.40, -0.21) | <.001 |
|  |  |  |
| Non-use | Ref |  |
| SSRIs | -0.39 (-0.51, -0.27) | <.001 |
| SNRIs | 0.28 (-0.32, 0.88) | 0.361 |
| TCAs | 0.24 (-0.29, 0.77) | 0.377 |
| Others | -0.23 (-0.40, -0.07) | 0.005 |
|  |  |  |
| Non-use | Ref |  |
| Citalopram (SSRI) | -0.40 (-0.55, -0.26) | <.001 |
| Sertraline (SSRI) | -0.28 (-0.48, -0.07) | 0.008 |
| Escitalopram (SSRI) | -0.74 (-1.09, -0.38) | <.001 |
| Venlafaxine (SNRI) | 0.04 (-0.74, 0.75) | 0.992 |
| Amitriptyline (TCA) | 0.24 (-0.29, 0.77) | 0.377 |
| Mirtazapine (Other) | -0.23 (-0.40, -0.07) | 0.005 |
|  |  |  |
| SSRIs | Ref |  |
| Others | 0.16 (-0.01, 0.34) | 0.067 |
|  |  |  |
| Sertraline (SSRI) | Ref |  |
| Citalopram (SSRI) | 0.29 (0.18, 0.40) | <.001 |
| Escitalopram (SSRI) | -0.42 (-84, 0.00) | 0.051 |
| Mirtazapine (Other) | 0.06 (-0.19, 0.31) | 0.645 |
| Abbreviations: CIs, confidence intervals; SSRIs, selective serotonin reuptake inhibitors; SNRIs, serotonin and norepinephrine reuptake inhibitors; TCAs, Tricyclic antidepressants; Others, other antidepressants; MMSE, Mini-Mental State Examination. | | |
| Adjused for age, sex, calendar year of diagnosis, the type of dementia, MMSE score at diagnosis, coresident status, care unit, depression, fracture, Charlson Comorbidity Index score, medications (angiotensin-converting enzyme inhibitors (ACEIs)/angiotensin receptor blockers (ARBs), β-blocking agents, calcium channel blockers, nonsteroidal anti-inflammatory drugs, diuretics, lipid modifying agents, antiplatelets, antipsychotics, anxiolytics and hypnotics). | | |

| Table S11. Stratified analysis of associations between antidepressants and cognitive decline (change in cognition slope, per y) by dementia medications in patients with AD and mixed dementia and LBD from SveDem, 2007-2018 | | |
| --- | --- | --- |
| Variables | AD and mixed | LBD |
|  | (n= 11 415) | (n=587) |
|  | β (95%CIs) | |
| ChEIs use | -0.29 (-0.43, -0.16) | 0.01 (-0.61, 0.63) |
| ChEIs non-use | -0.30 (-0.55, -0.05) | -0.52 (-1.83, 0.79) |
| Memantine use | -0.16 (-0.35, 0.04) | 0.64 (-0.19, 1.47) |
| Memantine non-use | -0.30 (-0.45, -0.15) | -0.85 (-1.64, -0.06) |
| Abbreviations: SveDem, the Swedish Registry for Cognitive/Dementia Disorders; CIs, confidence intervals; AD, Alzheimer’s disease; Mixed, mixed dementia; LBD, parkinson’s disease with dementia and dementia with Lewy bodies. | | |
| Adjused for age, sex, calendar year of diagnosis, the type of dementia, Mini-Mental State Examination score at diagnosis, coresident status, care unit, depression, fracture, Charlson Comorbidity Index score, medications (angiotensin-converting enzyme inhibitors (ACEIs)/angiotensin receptor blockers (ARBs), β-blocking agents, calcium channel blockers, nonsteroidal anti-inflammatory drugs, diuretics, lipid modifying agents, antiplatelets, antipsychotics, anxiolytics, hypnotics, cholinesterase inhibitors and memantine). | | |

| Table S12. Hazard ratios for adverse outcomes by antidepressant class and dose in patients from SveDem, 2007-2018 | | | |
| --- | --- | --- | --- |
| Antidepressant class and dose category | Severe dementia | All-cause mortality | Fracture |
|  | Hazard ratio (95% CIs) | | |
| Antidepressant class (binary) |  |  |  |
| Non-use | REF | REF | REF |
| Antidepressants | 1.06 (0.90-1.25) | 1.07 (1.01-1.13) | 1.18 (1.10-1.26) |
| Non-use | REF | REF | REF |
| SSRIs | 1.17 (0.98-1.41) | 1.05 (0.99-1.12) | 1.25 (1.15-1.35) |
| Non-use | REF | REF | REF |
| Others | 0.87 (0.67-1.13) | 1.10 (1.00-1.20) | 1.06 (0.94-1.19) |
|  |  |  |  |
| Antidepressant dose  (continuous and category) |  |  |  |
| Antidepressants (continuous) | 1.24 (1.04-1.47) | 1.13 (1.07-1.20) | 1.21 (1.12-1.31) |
|  |  |  |  |
| Non-use | REF | REF | REF |
| Antidepressants ≤0.5 DDD | 0.82 (0.63-1.07) | 0.98 (0.90-1.07) | 1.13 (1.02-1.25) |
| Antidepressants >0.5/≤1.0 DDD | 1.17 (0.94-1.45) | 1.06 (0.99-1.14) | 1.20 (1.09-1.32) |
| Antidepressants >1.0 DDD | 1.25 (0.96-1.62) | 1.19 (1.09-1.31) | 1.22 (1.08-1.38) |
|  |  |  |  |
| SSRIs (continuous) | 1.35 (1.12-1.62) | 1.11 (1.04-1.19) | 1.25 (1.15, 1.36) |
|  |  |  |  |
| Non-use | REF | REF | REF |
| SSRIs ≤ 0.5 DDD | 0.81 (0.56-1.17) | 1.01 (0.90-1.14) | 1.22 (1.06-1.39) |
| SSRIs >0.5/≤ 1.0 DDD | 1.33 (1.04-1.70) | 0.99 (0.91-1.08) | 1.26 (1.13-1.41) |
| SSRIs >1.0 DDD | 1.35 (1.02-1.80) | 1.18 (1.07-1.31) | 1.25 (1.10-1.43) |
|  |  |  |  |
| Others (continuous) | 0.86 (0.57-1.30) | 1.17 (1.04-1.32) | 1.04 (0.88-1.24) |
|  |  |  |  |
| Non-use | REF | REF | REF |
| Others ≤ 0.5 DDD | 0.84 (0.57-1.22) | 0.95 (0.83-1.09) | 1.07 (0.91-1.25) |
| Others >0.5/≤ 1.0 DDD | 0.87 (0.57-1.31) | 1.23 (1.08-1.39) | 1.07 (0.89-1.29) |
| Others >1.0 DDD | 0.88 (0.45-1.70) | 1.19 (0.97-1.47) | 0.99 (0.74-1.32) |
| Abbreviations: SveDem, the Swedish Registry for Cognitive/Dementia Disorders; CIs, confidence intervals; SSRIs, selective serotonin reuptake inhibitors; Others, other antidepressants. | | | |
| Adjusted for age, sex, calendar year of diagnosis, the type of dementia, Mini-Mental State Examination score at diagnosis, coresident status, care unit, depression, fracture, Charlson Comorbidity Index score, medications (angiotensin-converting enzyme inhibitors (ACEIs)/angiotensin receptor blockers (ARBs), β-blocking agents, calcium channel blockers, nonsteroidal anti-inflammatory drugs, diuretics, lipid modifying agents, antiplatelets, antipsychotics, anxiolytics and hypnotics). | | | |

| Table S13. Incidence Rate for adverse outcomes by antidepressant class in patients from SveDem, 2007-2018 | | | |
| --- | --- | --- | --- |
| Antidepressant class | Person-years | N | IR |
| Severe dementia |  |  |  |
| Non-use | 23300.3 | 531 | 22.8 |
| Antidepressants | 8309.5 | 208 | 25.0 |
| SSRIs | 5331.2 | 148 | 27.8 |
| Others | 2782.7 | 56 | 20.1 |
|  |  |  |  |
| All-cause mortality |  |  |  |
| Non-use | 60896.4 | 5947 | 97.7 |
| Antidepressants | 19476.7 | 1808 | 92.8 |
| SSRIs | 12886.4 | 1190 | 92.3 |
| Others | 6187.9 | 589 | 95.2 |
|  |  |  |  |
| Fracture |  |  |  |
| Non-use | 36982.2 | 3378 | 91.3 |
| Antidepressants | 11539.8 | 1224 | 106.1 |
| SSRIs | 7566.3 | 841 | 111.2 |
| Others | 3700 | 363 | 98.1 |
| Abbreviations: SveDem, the Swedish Registry for Cognitive/Dementia Disorders; CIs, confidence intervals; SSRIs, selective serotonin reuptake inhibitors; Others, other antidepressants; IR, incidence rate per 1 000 person-years. | | | |
| Adjused for age, sex, calendar year of diagnosis, the type of dementia, Mini-Mental State Examination score at diagnosis, coresident status, care unit, depression, fracture, Charlson Comorbidity Index score, medications (angiotensin-converting enzyme inhibitors (ACEIs)/angiotensin receptor blockers (ARBs), β-blocking agents, calcium channel blockers, nonsteroidal anti-inflammatory drugs, diuretics, lipid modifying agents, antiplatelets, antipsychotics, anxiolytics and hypnotics). | | | |

| Table S14. Incidence rate for adverse outcomes by antidepressant class and dose in patients from SveDem, 2007-2018 | | | | | | | | | |
| --- | --- | --- | --- | --- | --- | --- | --- | --- | --- |
| Antidepressant class and dose category | Severe dementia | | | All-cause mortality | | | Fracture | | |
|  | Person-years | N | IR | Person-years | N | IR | Person-years | N | IR |
| Antidepressants ≤0.5 DDD | 2568.6 | 58 | 22.6 | 5986.4 | 546 | 91.2 | 3718.1 | 415 | 111.6 |
| Antidepressants >0.5/≤1.0 DDD | 3364.6 | 93 | 27.6 | 7906.8 | 747 | 94.5 | 4564.8 | 505 | 110.6 |
| Antidepressants >1.0 DDD | 2376.3 | 57 | 24.0 | 5583.4 | 515 | 92.2 | 3256.9 | 304 | 93.3 |
|  |  |  |  |  |  |  |  |  |  |
| SSRIs ≤ 0.5 DDD | 1250.5 | 30 | 24.0 | 2972.9 | 288 | 96.9 | 1777.4 | 217 | 122.1 |
| SSRIs >0.5/≤ 1.0 DDD | 2235 | 72 | 32.2 | 5439 | 495 | 91.0 | 3163.6 | 368 | 116.3 |
| SSRIs >1.0 DDD | 1845.8 | 46 | 24.9 | 4474.6 | 407 | 91.0 | 2625.3 | 256 | 97.5 |
|  |  |  |  |  |  |  |  |  |  |
| Others ≤ 0.5 DDD | 1135 | 24 | 21.1 | 2632 | 232 | 88.1 | 1673.6 | 178 | 106.4 |
| Others >0.5/≤ 1.0 DDD | 1118.4 | 21 | 18.8 | 2451.3 | 250 | 102.0 | 1394.7 | 137 | 98.2 |
| Others >1.0 DDD | 529.4 | 11 | 20.8 | 1104.6 | 107 | 96.9 | 631.6 | 48 | 76.0 |
| Abbreviations: SveDem, the Swedish Registry for Cognitive/Dementia Disorders; SSRIs, selective serotonin reuptake inhibitors; Others, Other antidepressants; IR, incidence rate per 1 000 person-years. | | | | | | | | | |

| Table S15. Incidence Rate and hazard ratios for adverse outcomes by antidepressant class and dementia subtypes in patients from SveDem, 2007-2018 | | | | | | | | |
| --- | --- | --- | --- | --- | --- | --- | --- | --- |
| Antidepressant class | AD and Mixed dementia ^a^ | | | | VaD | | | |
|  | Person-years | N | IR | Hazard ratio  (95% CIs) | Person-years | N | IR | Hazard ratio  (95% CIs) |
| Severe dementia |  |  |  |  |  |  |  |  |
| Non-use | 13604.9 | 329 | 24.2 | REF | 2884 | 36 | 12.5 | REF |
| Antidepressants | 4894.6 | 137 | 28.0 | 1.11 (0.90-1.36) | 1037 | 20 | 19.3 | 1.30 (0.73-2.32) |
| SSRIs | 3246 | 99 | 30.5 | 1.23 (0.98-1.54) | 632 | 13 | 20.6 | 1.33 (0.68-2.62) |
| Others | 1548.1 | 37 | 23.9 | 0.84 (0.62-1.14) | 367 | 5 | 13.6 | 0.95 (0.34-2.65) |
|  |  |  |  |  |  |  |  |  |
| All-cause mortality |  |  |  |  |  |  |  |  |
| Non-use | 38072.5 | 3537 | 92.9 | REF | 6554.3 | 689 | 105.1 | REF |
| Antidepressants | 12075 | 1052 | 87.1 | 1.07 (1.00-1.15) | 2210.5 | 230 | 104.0 | 1.07 (0.93-1.24) |
| SSRIs | 8255.8 | 728 | 88.2 | 1.06 (0.98-1.15) | 1367.9 | 142 | 103.8 | 1.07 (0.90-1.27) |
| Others | 3609.7 | 315 | 87.3 | 1.12 (0.99-1.26) | 772.4 | 80 | 103.6 | 1.05 (0.83-1.32) |
|  |  |  |  |  |  |  |  |  |
| Fracture |  |  |  |  |  |  |  |  |
| Non-use | 23355.6 | 2012 | 86.1 | REF | 4008 | 402 | 100.3 | REF |
| Antidepressants | 7237.9 | 720 | 99.5 | 1.18 (1.08-1.29) | 1350.5 | 163 | 120.7 | 1.17 (0.98-1.41) |
| SSRIs | 4844.5 | 509 | 105.1 | 1.25 (1.13-1.38) | 835.7 | 109 | 130.4 | 1.30 (1.05-1.62) |
| Others | 2242.3 | 200 | 89.2 | 1.04 (0.89-1.21) | 468.6 | 48 | 102.4 | 0.96 (0.70-1.32) |
| Abbreviations: SveDem, the Swedish Registry for Cognitive/Dementia Disorders; CIs, confidence intervals; AD, Alzheimer’s disease; Mixed, mixed dementia; VaD, vascular dementia; SSRIs, selective serotonin reuptake inhibitors; Others, other antidepressants; IR, incidence rate per 1 000 person-years. | | | | | | | | |
| Adjused for age, sex, calendar year of diagnosis, the type of dementia, Mini-Mental State Examination score at diagnosis, coresident status, care unit, depression, fracture, Charlson Comorbidity Index score, medications (angiotensin-converting enzyme inhibitors (ACEIs)/angiotensin receptor blockers (ARBs), β-blocking agents, calcium channel blockers, nonsteroidal anti-inflammatory drugs, diuretics, lipid modifying agents, antiplatelets, antipsychotics, anxiolytics and hypnotics).  ^a^, further adjusted for cholinesterase inhibitors and memantine. | | | | | | | | |
